# Opportunistic Genomic Screening for Familial Hypercholesterolemia to Improve Low-Density Lipoprotein Cholesterol: A Randomized Clinical Trial

**DOI:** 10.1001/jamanetworkopen.2025.49664

**Published:** 2026-01-09

**Authors:** Jason L. Vassy, Charles A. Brunette, Thomas Yi, Themistocles L. Assimes, Kurt D. Christensen, Joshua W. Knowles, Amy C. Sturm, Yan V. Sun, Nicholas Alexander, Mark P. Cardellino, Alicia Harrison, Haley L. Gerety, Mary Pyatt, Ron Vered, Peter W. F. Wilson, Pradeep Natarajan, Stacey B. Whitbourne, J. Michael Gaziano, Sumitra Muralidhar, Morgan E. Danowski

**Affiliations:** 1 Veterans Affairs (VA) Boston Healthcare System, Boston, Massachusetts; 2 Harvard Medical School, Boston, Massachusetts; 3 VA Palo Alto Healthcare System, Palo Alto, California; 4 Department of Medicine, Stanford University School of Medicine, Stanford, California; 5 Precision Medicine Translational Research Center, Department of Population Medicine, Harvard Pilgrim Health Care Institute, Boston, Massachusetts; 6 Division of Cardiovascular Medicine, Cardiovascular Institute, Department of Medicine, Stanford University School of Medicine, Stanford, California; 7 Cardiovascular Institute, Department of Medicine, Stanford University School of Medicine, Stanford, California; 8 Prevention Research Center, Stanford University School of Medicine, Stanford, California; 9 Diabetes Research Center, Stanford University School of Medicine, Stanford, California; 10 The Family Heart Foundation, Fernandina Beach, Florida; 11 Ohio State Genomic Health, The Ohio State University Wexner Medical Center, Columbus, Ohio; 12 Atlanta VA Healthcare System, Atlanta, Georgia; 13 Department of Epidemiology and Global Health, Emory University Rollins School of Public Health; 14 Bucharest University for Economic Studies, Bucharest, Romania; 15 Emory Clinical Cardiovascular Research Institute, Atlanta, Georgia; 16 Center for Genomic Medicine and Cardiovascular Research Center, Massachusetts General Hospital, Boston; 17 Program in Medical and Population Genetics, Broad Institute of Harvard and MIT, Boston, Massachusetts; 18 Division of Aging, Brigham and Women’s Hospital, Boston, Massachusetts; 19 Veterans Health Administration, Office of Research and Development, Washington, DC

## Abstract

**Importance:**

The clinical benefit of opportunistic genomic screening for familial hypercholesterolemia (FH) has not been demonstrated in a randomized clinical trial (RCT).

**Objective:**

To evaluate the impact of returning clinically confirmed FH-associated genetic results on low-density lipoprotein cholesterol (LDL-C) levels.

**Design, Setting, and Participants:**

This RCT was performed within the Veterans Health Administration, a large national health care system, and linked to the Million Veteran Program (MVP), a research biobank. Participants were MVP enrollees suspected to have an FH-associated genetic variant, as identified in their research data. Recruitment occurred from February 27, 2020, to September 20, 2022, and 6-month follow-up was completed October 21, 2024.

**Interventions:**

Delivery of clinical genetic confirmation testing and telegenetic counseling at baseline (immediate results arm) vs after 6 months (delayed results arm).

**Main Outcomes and Measures:**

Change in LDL-C levels (primary outcome) and proportions with treatment intensification and achievement of LDL-C target levels at 6 months (secondary outcomes).

**Results:**

The trial randomized 112 participants across 28 US states (mean age, 65.9 [range, 36-91] years; 94 [83.9%] men). Baseline mean (SD) LDL-C level was 109.5 (55.5) mg/dL, and 86 participants (76.8%) were already receiving therapy to lower lipid levels. At 6 months, the between-arm difference in LDL-C level reduction was −10.5 (95% CI, −21.9 to 1.0) mg/dL (*P* = .07; Cohen *d* = 0.34). Bayesian analysis suggested a high probability of benefit but was exploratory. Treatment was intensified in 11 of 55 participants (20.0%) in the immediate results arm vs 5 of 57 (8.8%) in the delayed results arm (*P* = .09). Fifteen participants (27.3%) in the immediate results arm vs 14 (24.6%) in the delayed results arm (*P* = .74) achieved LDL-C target levels. Thirty of 49 participants (61.2%) in the immediate results arm who completed this information shared their genetic result with a total of 98 relatives.

**Conclusions and Relevance:**

In this RCT, opportunistic genomic screening for FH plus telegenetic counseling did not result in a statistically significant improvement in LDL-C levels and clinical management; however, the findings suggest that there may be a small to moderate benefit favoring the immediate results arm. Further research should be conducted to confirm these findings, optimize implementation strategies, and assess the long-term effects on cardiovascular outcomes.

**Trial Registration:**

ClinicalTrials.gov Identifier: NCT04178122

## Key Points

**Question:**

Does opportunistic genomic screening for familial hypercholesterolemia (FH) reduce low-density lipoprotein cholesterol (LDL-C) levels in a national health care system?

**Findings:**

In this randomized clinical trial of 112 participants suspected to have FH-associated genetic variants, participants who received immediate disclosure of their results and telegenetic counseling had a 10.5-mg/dL greater reduction in LDL-C levels at 6 months than those with delayed disclosure. This difference was not statistically significant, but an exploratory bayesian analysis suggested a high probability of benefit for the intervention.

**Meaning:**

These findings suggest that opportunistic genomic screening for FH may modestly improve LDL-C levels, even in populations already receiving therapy to lower lipid levels, but results should be confirmed in larger studies.

## Teaser

This randomized clinical trial investigates whether opportunistic genomic screening for familial hypercholesterolemia was effective in reducing low-density lipoprotein cholesterol (LDL-C) levels among participants in the Million Veteran Program.

## Introduction

Opportunistic genomic screening—the identification of clinically actionable genetic variants in individuals undergoing genetic testing for unrelated reasons—has become increasingly feasible as sequencing technologies advance and testing costs decline.^1,2^ In parallel, professional organizations have recommended returning pathogenic variants associated with certain actionable monogenic conditions.^3,4,5^ Although the promise of genomic screening to identify individuals at risk for serious but preventable diseases is compelling,^6,7,8,9^ no randomized clinical trial (RCT) has demonstrated improvements in clinical outcomes or intermediate risk factor levels from such an approach.

Familial hypercholesterolemia (FH) represents a model condition for evaluating the clinical utility of genomic screening. FH is a common genetic disorder that leads to elevated low-density lipoprotein cholesterol (LDL-C) levels and a substantially increased risk of premature cardiovascular disease.^10^ Despite the availability of effective therapies to lower lipid levels, FH remains underdiagnosed and undertreated.^10,11,12^ Most existing FH screening efforts have focused on diagnostic testing in individuals with clinically suspected FH and cascade testing in families of known cases—strategies shown to improve outcomes and be cost-effective.^10,13,14^ In contrast, opportunistic genomic screening for FH in unselected populations—sometimes described as a genotype-first approach—has not been evaluated in an RCT, and its potential impact on cardiovascular risk reduction remains uncertain.^6,7,8^ Rigorous evidence of benefit is needed to inform whether health care systems or public health programs should invest resources in genomic screening as a population health strategy.

The Million Veteran Program (MVP) Return of Actionable Results (ROAR) Study was designed to generate RCT evidence on the impact of identifying and returning FH-associated genetic results to patients and their clinicians within a large national integrated health care system.^15^ This study aimed to inform the role of genomic screening in clinical medicine by testing the hypothesis that returning FH-associated genetic results leads to improved clinical management and outcomes, as defined by intensified therapy to lower lipid levels and lower LDL-C values.

## Methods

### Trial Design and Participants

The MVP-ROAR Study was an RCT designed to evaluate the impact of returning clinically confirmed FH-associated genetic results on lipid management within the Veterans Health Administration of the US Department of Veterans Affairs (VA). The trial protocol has been described previously^15^ and is found in Supplement 1. The VA Central Institutional Review Board approved this study; all participants gave verbal informed consent. This study followed the Consolidated Standards of Reporting Trials (CONSORT) reporting guideline for RCTs.

The VA is a nationwide integrated health care system that provides care to more than 9 million US military veterans at more than 1300 health care facilities in all US states and territories.^16,17^ Trial participants were recruited from the MVP, a national biobank that links genomic, survey, and electronic health record data from more than 1 million veterans to date.^18^ At the time recruitment began, 461 590 MVP participants had genotype data available for analysis.

### Eligibility Criteria

Participants were eligible for the study if they were living MVP participants with a suspected pathogenic or likely pathogenic variant identified through the MVP research genotyping array in 1 of 4 FH-associated genes: low-density lipoprotein receptor (*LDLR*), apolipoprotein B (*APOB*), low-density lipoprotein receptor adapter protein 1 (*LDLRAP1*), and proprotein convertase subtilisin/kexin type 9 (*PCSK9*).^19,20^ Individuals were excluded if their medical record documented a prior molecular diagnosis of FH.^15^

### Recruitment and Enrollment

Recruitment began on February 27, 2020, and proceeded in batches of invitations as variants from the MVP array were determined to be eligible for confirmation and return. Eligible participants were initially contacted by mail with an introductory letter describing the study. Individuals who did not opt out were subsequently contacted by telephone by a genetic counselor (M.E.D.), who provided detailed study information, confirmed eligibility, and obtained informed consent. Consenting participants completed a baseline survey and provided clinical biospecimens for baseline LDL-C measurements and for confirmatory gene panel sequencing in a clinical laboratory for the suspected FH-associated variant. During the COVID-19 pandemic, study procedures were amended to allow at-home saliva collection for DNA. Recruitment ended on September 20, 2022, before meeting the target sample size. Follow-up for all participants was completed on October 21, 2024.

### Clinical Confirmation of Research Variants

Participant DNA specimens from the MVP biobank undergo genotyping on the MVP 1.0 custom Axiom array (Applied Biosystems), which includes 668 418 genetic markers passing quality control and selected for representation across genetic ancestry groups.^21^ As described previously,^15^ the study only considered for return those variants in *LDLR*, *APOB*, *LDLRAP1*, and *PCSK9* that were directly genotyped on the MVP array and were classified as pathogenic or likely pathogenic according to American College of Medical Genetics and Genomics and the Association for Molecular Pathology criteria^22^; the study ultimately reached out only to participants suspected to carry variants in *LDLR* and *APOB*. Variants were restricted to those with 3- or 4-star interpretations in ClinVar. Each variant considered for return was additionally curated with input from the ClinGen FH Variant Curation Expert Panel^19,20^ and with variant curation experts at the confirming clinical laboratory (Invitae Corporation) before study staff approval for return and selection of eligible participants. The new DNA specimen from each enrollee was shipped to a Clinical Laboratory Improvement Amendments (CLIA)–certified laboratory (Invitae Corporation) for clinical confirmation of the research variant using a targeted gene panel.^15^ As previously described, during an initial pilot study, suspected FH-associated variant research results were not confirmed on clinical sequencing for 3 of 8 participants, prompting implementation of an additional quality control method for calling rare heterozygous genotypes from the MVP array.^15,23^

### Intervention

After DNA specimen receipt, participants were randomly assigned in a 1:1 ratio to 1 of 2 study arms using a computer-generated permuted block randomization scheme with block sizes of 4.^15^ Participants in the immediate results arm received the results of their clinical genetic confirmatory testing and a telegenetic counseling intervention at baseline. Participants in the delayed results arm received the same intervention 6 months after randomization, following outcome data collection.

The intervention consisted of a brief telegenetic counseling session conducted by a board-certified genetic counselor (M.E.D.) via telephone or videoconferencing. The session included disclosure of the gene panel sequencing results, explanation of the inheritance pattern and associated cardiovascular risks of any positive results, and review of guideline-based treatment recommendations. The counselor reviewed the participant’s LDL-C value and discussed the benefits of family cascade screening. Participants received educational materials, a personalized summary letter, and a letter to share with relatives. Results and clinical guidance were also sent to the participant’s primary care clinician.^15^

### Data Collection and Outcomes

As previously detailed, data collection included study LDL-C level measurements, participant surveys at baseline and 6 months, and medical record review.^15^ Participants self-reported race and ethnicity on the baseline survey, including American Indian or Alaska Native, Asian, Black or African American, Hispanic or Latino, White, multiracial, or other race or ethnicity; these data were collected to evaluate the burden of pathogenic variants among populations of different backgrounds. The primary outcome was change in LDL-C level at 6 months, calculated as the difference between the 6-month and baseline values. During the COVID-19 pandemic, study procedures were amended to allow for the use of extant clinical LDL-C values within 6 months prior to enrollment as the baseline measurement. For participants for whom a 6-month study blood draw was not feasible, the closest clinical LDL-C value from the medical record was used as the end-of-study measurement if within 360 days from randomization. In the absence of a study or suitable clinical end-of-study LDL-C level measurement, baseline LDL-C level was carried forward for analysis. The 2 secondary outcomes were: (1) proportion of participants meeting LDL-C target levels at 6 months, defined as LDL-C level of less than 100 mg/dL (to convert to mmol/L, multiply by 0.0259) for primary prevention and LDL-C level of less than 70 mg/dL for secondary prevention; and (2) intensification of therapy to lower lipid levels, defined as initiation of a new medication, dose escalation of an existing medication, or addition of a second agent. Secondary prevention was defined as any patient with pre-existing atherosclerotic disease (ASCVD) or presence of any ASCVD risk factor, including 65 years or older, prior percutaneous coronary intervention, prior coronary artery bypass graft, other evidence of coronary artery disease, diabetes, hypertension, chronic kidney disease, current smoking, congestive heart failure, family history of premature ASCVD, ankle-brachial index less than 0.9, elevated coronary artery calcium score, or elevated levels of lipoprotein A or apolipoprotein B. Intensification of pharmacotherapy at 6 months was a composite outcome including prescription of new monotherapy, dose escalation of existing pharmacotherapy, or addition of 1 or more medications to existing pharmacotherapy compared with baseline pharmacotherapy status. Exploratory outcomes included medication adherence, defined as proportion of days covered greater than 80%, based on pharmacy data^24^; cascade testing among first-degree relatives; lifestyle behaviors, including smoking, physical activity, and saturated fat intake; and health-related quality of life.^15^ Outcome assessors were blinded to study arm assignment.

### Statistical Analysis

Primary and secondary outcomes were compared between groups as described in the statistical analysis plan in Supplement 1.^15^ Outcomes were analyzed using both an intention-to-treat approach among all randomized participants and a per-protocol approach among participants who received a positive clinical genetic test result for an FH-associated variant (eTable 1 in Supplement 2). The statistical software R, version 4.2.2 (R Project for Statistical Computing) was used to analyze the data.

The study aimed to enroll 244 participants to enable 80% power to detect a between-group difference of 20% in 6-month change in LDL-C levels at a 2-sided α = .05, as described previously.^15^ Due to COVID-19–related disruptions and funding constraints, the trial did not meet its planned sample size. A post hoc bayesian analysis was thus conducted to estimate the probability of benefit under the assumption that the observed effect would have persisted had the full sample size been achieved. This analysis estimated the posterior distribution of the between-group difference in LDL-C levels using noninformative priors and normal likelihoods. Exploratory outcomes were analyzed as described previously.^15^

## Results

### Participant Characteristics

**Figure 1.**
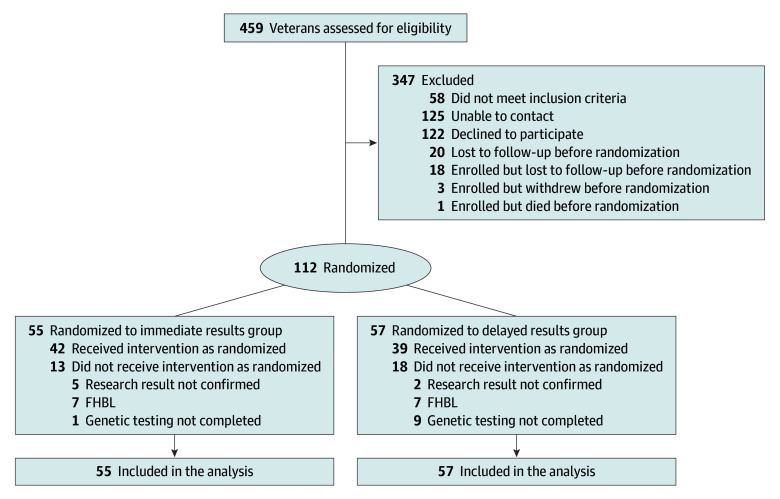
Study Flow Diagram for the Million Veteran Program–Return of Actionable Results Study FHBL indicates familial hypobetalipoproteinemia.

**Figure 2.**
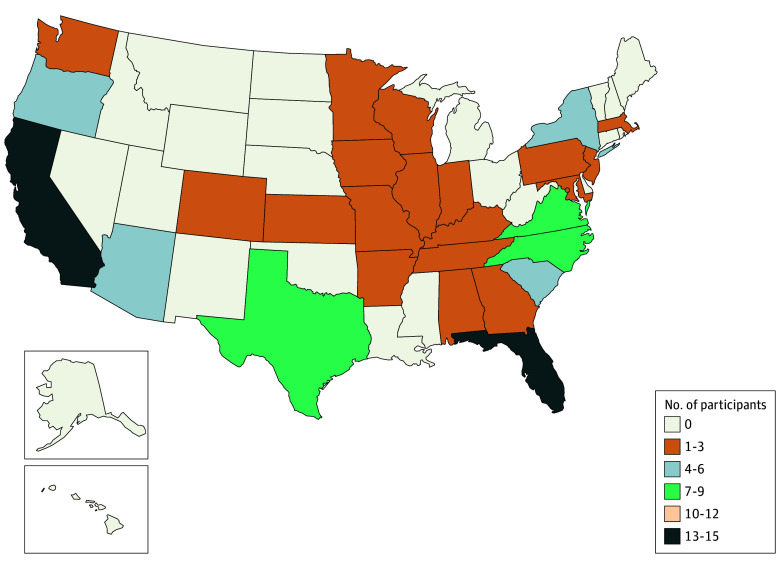
State of Residence of Million Veteran Program–Return of Actionable Results Study Enrollees

Of 459 MVP participants with a suspected FH-associated variant invited to participate, 134 enrolled in the trial and 112 completed baseline DNA biospecimen collection and were ultimately randomized (mean age, 65.9 [range, 13.3-36-91] years; 94 [83.9%] men and 18 [16.1%] women) (Table 1). A study flow diagram is presented in Figure 1. The 112 randomized participants resided in 28 US states (Figure 2); 48 (42.9%) resided in moderately or highly disadvantaged areas of the US, as measured by Area Deprivation Index (Table 1). Most participants were Black or African American (31 [27.7%]) or White (72 [64.3%), while smaller proportions were American Indian or Alaska Native (1 [0.9%]), Asian (2 [1.8%]), multiracial (3 [2.7%]), and of other (1 [0.9%]) or unknown (2 [1.8%]) race; 10 participants (8.9%) reported Hispanic or Latino ethnicity. The mean maximum historical LDL-C values recorded in the electronic health record for trial enrollees was 190.2 (range, 39.0-480.0) mg/dL. At trial baseline, 86 participants (76.8%) were taking therapy to lower lipid levels (Table 1) and 25 (22.3%) were meeting LDL-C level targets for primary or secondary prevention (Table 2).

**Table 1.** Baseline Characteristics of Million Veteran Program–Return of Actionable Results Study Trial Participants

Characteristic	Overall (N = 112)	Delayed disclosure of results (n = 57)	Immediate disclosure of results (n = 55)
Age, mean (SD), y	65.9 (13.3)	65.2 (12.6)	66.7 (14.1)
Sex, No. (%)			
Female	18 (16.1)	7 (12.3)	11 (20.0)
Male	94 (83.9)	50 (87.7)	44 (80.0)
Race, No. (%)			
American Indian or Alaska Native	1 (0.9)	1 (1.8)	0
Asian^a^	2 (1.8)	1 (1.8)	1 (1.8)
Black or African American	31 (27.7)	19 (33.3)	12 (21.8)
White	72 (64.3)	33 (57.9)	39 (70.9)
Multiracial	3 (2.7)	1 (1.8)	2 (3.6)
Other^b^	1 (0.9)	0	1 (1.8)
Unknown	2 (1.8)	2 (3.5)	0
Ethnicity, No. (%)			
Hispanic or Latino	10 (8.9)	6 (10.5)	4 (7.3)
Not Hispanic or Latino	101 (90.2)	50 (87.7)	51 (92.7)
Unknown	1 (0.9)	1 (1.8)	0
Area Deprivation Index, No. (%)^c^			
Least disadvantaged	48 (42.9)	26 (45.6)	22 (40.0)
Moderately disadvantaged	38 (33.9)	19 (33.3)	19 (34.5)
Most disadvantaged	26 (23.2)	12 (21.1)	14 (25.5)
Maximum LDL-C in EHR, mean (SD) [range], mg/dL^d^	190.2 (80.8) [39 to 480]	192.6 (83.5) [48 to 480]	187.6 (78.7) [39 to 454]
Lipid-lowering pharmacotherapy, No. (%)	86 (76.8)	49 (86.0)	37 (67.3)

Abbreviations: EHR, electronic health record; LDL-C, low-density lipoprotein cholesterol.

SI conversion factor: To convert cholesterol to mmol/L, multiply by 0.0259.

^a^ Includes Asian Indian, Chinese, Filipino, Japanese, and other Asian race.

^b^ Participants selecting this category on the baseline survey were not asked to specify further.

^c^ Categorized as least disadvantaged (1-40), moderately disadvantaged (41-70), and most disadvantaged (71-100).

^d^ Includes all randomized participants; maximum observed LDL-C level for 81 participants in per-protocol analyses were a mean of 208.1 (range, 102-454) mg/dL.

**Table 2.** Six-Month LDL Cholesterol Level and Therapy to Lower Lipid Level Outcomes Among Million Veteran Program–Return of Actionable Results Study Trial Participants

Measurement	Overall (N = 112)	Immediate disclosure of results (n = 55)	Delayed disclosure of results (n = 57)	Between-arm difference, (95% CI)	Between-arm comparison^a^
Baseline					
LDL-C level, mean (SD) [range], mg/dL^b^	109.5 (55.5) [2 to 376]	114.4 (64.1) [22 to 376]	104.7 (45.7) [2 to 274]	9.7 (−11.1 to 30.5)	NA
Meeting LDL-C target level, No. (%)	25 (22.3)	11 (20.0)	14 (24.6)	−0.05 (−0.20 to 0.11)	NA
6 mo					
LDL-C level, mean (SD) [range], mg/dL^c^	106.8 (58.2) [2 to 444]	106.4 (69.0) [8 to 444]	107.2 (46.2) [2 to 229]	−0.8 (−22.7 to 21.2)	NA
Meeting LDL-C target level, No. (%)^d^	29 (25.9)	15 (27.3)	14 (24.6)	0.03 (−0.14 to 0.19)	*P* = .74; Cohen *h* = 0.06
ΔLDL-C, mean (SD) [range], mg/dL	−2.7 (30.9) [−129 to 68]	−8.0 (32.3) [−129 to 68]	2.5 (28.9) [−123 to 49]	−10.5 (−21.9 to 1.0)	*P* = .07^e^; Cohen *d* = 0.34
Intensification of therapy to lower lipid levels, No. (%)	16 (14.3)	11 (20.0)	5 (8.8)	0.11 (−0.02 to 0.24)	*P* = .09; Cohen *h* = 0.33

Abbreviations: LDL-C, low-density lipoprotein cholesterol; NA, not applicable.

^a^ Values correspond to *z* test of proportions and Cohen *h* and *d* values, as indicated, where 0.2 indicates small effect; 0.5, medium effect; and 0.8, large effect.

^b^ Derived from study blood draw or clinical value occurring on or before randomization date.

^c^ Derived from end-of-study blood draw, clinical value closest to end of 6-month observation period (by absolute value, not exceeding 360 days from randomization or occurring after results disclosure), or baseline value carried forward if no 6-month value available.

^d^ Defined as less than 70 mg/dL for secondary prevention or less than 100 mg/dL for primary prevention.

^e^ Calculated using a 2-sample *t* test (using absolute ΔLDL-C); similar results using analysis of covariance (*P* = .10), Wilcoxon rank sum (*P* = .08), and Bayesian analysis (posterior mean difference, −10.5 mg/dL; 95% credible interval, −21.7 to 1.0 mg/dL; 96.5% probability that the true mean difference in ΔLDL-C is lower [<0 mg/dL] in the immediate results arm [estimated mean, −8.0 mg/dL; sample SD, 30.6 mg/dL] than in the delayed results arm [estimated mean, 2.5 mg/dL; sample SD, 30.6 mg/dL]).

Among 98 pedigrees collected, 59 participants reported a family history of myocardial infarction or stroke, and 6 reported a personal history of myocardial infarction. No participant reported prior genetic testing for FH.

### Clinical Gene Sequencing Results

Of the 112 randomized participants, 102 completed confirmatory clinical gene panel testing. Among them, clinical sequencing confirmed the suspected MVP research variant in 94 participants. Fourteen of these participants had the *APOB* c.10238del (NM_000384.2) or *APOB* c.7537C>T (NM_000384.2) variant, which are associated with familial hypobetalipoproteinemia, a rare monogenic condition characterized by low LDL-C levels, distinct from FH. These participants were included in intention-to-treat analyses but excluded from per-protocol analyses focused on FH-associated variants (eTable 1 in Supplement 2).

In the remaining 8 participants, the suspected research variant was not confirmed by results of clinical testing. All of these cases occurred prior to the implementation of the improved rare variant calling algorithm.^15,23^ One participant was found to have a different pathogenic variant: *LDLR* c.1586 + 1G>A (NM_000527.4) in addition to the originally suspected *APOB* c.11273T>C (NM_000384.2); this individual was included in per-protocol analyses.

Confirmed results were also shared with participants’ health care clinicians, including 90 VA and 17 non-VA primary care clinicians.

### Primary Outcome

At baseline, the mean (SD) LDL-C level across both study arms was 109.5 (55.5) mg/dL (Table 2). At 6 months, the mean (SD) 6-month change in LDL-C level was −8.0 (32.3) mg/dL (95% CI, −16.7 to 0.7 mg/dL) in the immediate results arm (7.0% reduction) and 2.5 (28.9) mg/dL (95% CI −5.2 to 10.1 mg/dL) in the delayed results arm (2.4% increase). The between-arm difference in LDL-C reduction was −10.5 mg/dL (95% CI, −21.9 to 1.0 mg/dL; *P* = .07). The effect size (Cohen *d*) was 0.34, suggesting a small to moderate benefit favoring the immediate results arm, although the primary comparison did not reach statistical significance.

A post hoc bayesian analysis estimated a 96.5% posterior probability that the mean reduction in LDL-C level was greater in the immediate results arm than in the delayed results arm. Assuming the observed effect had persisted in the originally planned sample size (n = 244), the bayesian model estimated a 95% credible interval of −18.2 to −2.9 mg/dL and a 99.6% probability of benefit.

Per-protocol analyses were similar: the between-arm difference in LDL-C level reduction was −11.7 mg/dL (95% CI −26.8 to 3.4 mg/dL; *P* = .13; Cohen *d* = 0.34) (eTable 2 in Supplement 2). A sensitivity analysis excluding 15 participants with missing 6-month LDL-C values also yielded similar results, with a mean between-arm difference of −12.2 mg/dL (95% CI, −25.4 to 1.0 mg/dL; *P* = .07).

### Secondary Outcomes

At baseline, 86 participants (76.8%) were receiving therapy to lower lipid levels, but only 25 (22.3%) were meeting LDL-C target levels for primary or secondary prevention (Table 2). At 6 months, 15 participants (27.3%) in the immediate results arm and 14 (24.6%) in the delayed results arm were meeting their respective LDL-C targets (Cohen *h* = 0.06; *P* = .74).

**Figure 3.**
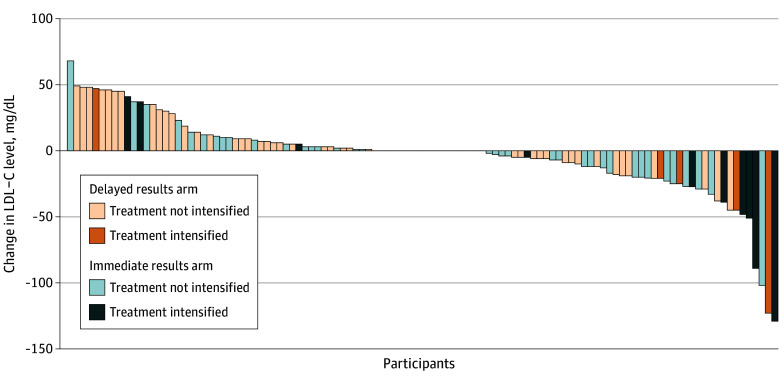
Individual 6-Month Change in Low-Density Lipoprotein Cholesterol (LDL-C) Levels by Study Arm and Intensification of Therapy to Lower Lipid Levels

Therapy to lower lipid levels was intensified in 16 participants (14.3%) overall: 11 of 55 (20.0%) in the immediate results arm and 5 of 57 (8.8%) in the delayed results arm (Cohen *d* = 0.33; *P* = .09). Details of prescription changes by study arm are presented in eTable 3 in Supplement 2. Notably, 56 participants (50.0%) were receiving therapy to lower lipid levels at baseline and did not undergo any treatment intensification during the study period. Figure 3 shows the distribution of individual 6-month LDL-C changes, stratified by study arm and treatment escalation status.

### Other Outcomes

Among participants in the immediate results arm, 30 of 49 who completed these survey items (61.2%) reported sharing their genetic results with at least 1 relative, for a total of 98 relatives (eTable 4 in Supplement 2). Three participants confirmed that a total of 4 relatives had undergone cascade genetic testing. At 6 months, 35 of 53 participants (66.0%) agreed that the study team should have contacted them directly about the research results, while 9 (17.0%) preferred that their primary care clinician be contacted first. Twenty-five of 49 participants (51.0%) preferred communication of clinical genetic results from the study’s genetic counselor; 12 (24.5%) preferred disclosure by their primary care clinician. Psychosocial responses, measured via the Feelings About Genomic Testing Results (FACToR) questionnaire, indicated low levels of negative emotions, uncertainty, and privacy concerns.^25^

There were no between-arm differences in 6-month change in health-related quality of life or patient activation (eTable 5 in Supplement 2). Six-month change in belief that medications are harmful did not differ between arms, but participants in the delayed results arm had greater improvement in belief that medications are overused compared with those in the immediate results arm (mean [SD] score, −0.89 [2.25] vs 0.11 [2.47]; *P* = .03; scores for the overuse subscale range from 4 to 20, with higher scores indicating a stronger belief that medications are overprescribed and overused) (eTable 5 in Supplement 2). Among 71 participants with complete pharmacy data, adherence to therapy to lower lipid levels was observed in 27 of 34 (79.4%) in the immediate results arm and 28 of 37 (75.7%) in the delayed results arm (*P* = .71). At 6 months, the distribution of participants across stages of behavioral change for increasing physical activity, smoking cessation, and reducing saturated fat intake did not differ between study arms (eTable 6 in Supplement 2).

## Discussion

In this RCT embedded in a national health care system linked to a megabiobank, returning clinically confirmed FH-associated genetic results to patients and clinicians was associated with a modest, nonsignificant reduction in LDL-C levels during follow-up of 6 months compared with delayed return. A 10-mg/dL reduction in LDL-C levels, similar to the observed between-group difference in this study, has been associated in prior meta-analyses with lower rates of coronary events and stroke, particularly among high-risk populations.^26,27^ While the primary outcome did not reach statistical significance, post hoc bayesian analysis estimated a high probability of benefit had the trial achieved its planned sample size. These findings must be interpreted cautiously given their exploratory nature, but they suggest that opportunistic genomic screening for FH may warrant further evaluation in clinical practice.

It is notable that two-thirds of participants were already using therapy to lower lipid levels at baseline, although only one-quarter were meeting LDL-C target levels. The study intervention—a single telegenetic counseling session with accompanying educational materials for patients and clinicians—was designed for clinical feasibility. Enrolling only patients not already using therapy or using a more protocolized treatment and monitoring intervention would likely have yielded larger effect sizes. The fact that LDL-C improvements were most pronounced among participants who underwent treatment intensification underscores the importance of clinical action in translating molecular diagnosis into improved cardiovascular health, on the part of both the clinicians who prescribe therapy and the patients who adhere to therapy. Indeed, interviews among primary care clinicians who received their patients’ FH results in this trial suggest that additional implementation strategies may be needed to drive change in clinical management.^28^

A clinical diagnosis of FH can be made through criteria including lipid level thresholds, family history, and physical examination findings.^29^ Among patients with a clinical FH diagnosis, genetic testing has been shown to promote further improvements in treatment and LDL-C levels.^10,30,31,32^ In contrast to the diagnostic testing context, many health care systems and research initiatives have launched opportunistic or population screening programs in unselected patient populations, including return of FH-associated variants.^6,7,9,33,34^ For example, the Geisinger genomic screening program has returned approximately 600 FH-associated results to patients to date^8^; an early analysis including the first 93 patients suggested that receipt of this information prompted new clinical FH diagnoses and electronic health record documentation.^33^ However, none of these programs has demonstrated that such programs improve LDL-C levels, an important determinant of cardiovascular risk.

No prior trial, to our knowledge, has demonstrated that return of genetic results can lower LDL-C levels among a population identified from a genotype-first approach. In 1 small trial in Japan, patients with clinically diagnosed FH who received genetic testing had a 20-mg/dL greater reduction in LDL-C levels at 6 months compared with waiting list controls.^32^ In the Myocardial Infarction Genes (MI-GENES) Study among patients at intermediate risk for coronary heart disease but not already using statin therapy, disclosure of risk estimates that incorporated genetic risk information led to greater statin prescriptions and lower LDL-C levels at 6 months compared with disclosure of conventional risk estimates.^35^ Unlike these 2 trials, the present study suggests that a genotype-first approach in a clinical, unselected population might lower LDL-C levels, although further research is needed to confirm clinical impact.

These results may inform health care systems about the implementation of genomic screening in routine clinical care. Although our trial focused on FH, its clinical testing and return pathways, condition-specific decision support, and outcomes assessments are readily transferable to other high-evidence conditions. Future work should prioritize screening for carefully curated variants in genes associated with high-actionability conditions and continue to develop evidence on downstream clinical utility. Economic modeling studies have suggested that population genomic screening might not be cost-effective for FH alone^36,37^ but might be when combined with genomic screening for hereditary breast and ovarian cancer and Lynch syndrome, 2 other prevalent, highly penetrant monogenic diseases.^38,39^ Expanding genomic screening to additional conditions such as cardiomyopathies and other hereditary cancer syndromes would increase the yield of screening but might reduce cost-effectiveness if included variants have lower penetrance or less well-established interventions.^3,40,41^ In any case, significant challenges to the implementation of genomic screening remain. Implementation at scale will require robust laboratory and informatics infrastructure, clear clinical workflows for result disclosure and management, and expanded capacity for genetic counseling. Ethical considerations—including data privacy, result interpretation, and potential for genetic discrimination—also warrant careful attention in future program design. Still, the present study contributes empirical data to this discussion, suggesting that return of FH-associated genetic results may influence lipid management even in a highly treated population.

### Strengths and Limitations

This study has several strengths, including its design as an RCT embedded within a large, integrated health care system and its use of LDL-C levels, a clinically important biomarker, to evaluate the impact of the intervention. The trial leveraged an existing biobank and electronic health record infrastructure to assess the clinical implementation of genomic result return.

Several limitations also warrant consideration. First, the study did not reach its planned enrollment target due to COVID-19 pandemic–related disruptions and funding constraints, limiting statistical power to detect small-to-moderate effects. Second, not all randomized participants received the intended intervention due to loss to follow-up, nonconfirmation of suspected variants, or identification of *APOB* variants associated with low LDL-C levels rather than FH. Nevertheless, intention-to-treat analyses that included these cases still showed consistent trends. Third, the study relied on variants detected through genotyping arrays, which may miss rarer pathogenic variants that require sequencing. Fourth, only 16.1% of participants were female; trial results may differ with inclusion of a greater proportion of women with FH, who are less likely to be treated intensively with therapy to lower lipid levels and to attain recommended LDL-C level targets.^42^ Finally, while LDL-C levels constitute a well-validated surrogate marker, they are not a direct measure of cardiovascular outcomes such as myocardial infarction or mortality.

## Conclusions

This study provides RCT data on the feasibility and short-term clinical impact of returning FH-associated genetic results within a national health care system. Although the primary outcome did not reach statistical significance, the findings suggest that opportunistic genomic screening may influence lipid management. Further research is needed to confirm these findings, optimize implementation strategies, and evaluate long-term effects on cardiovascular outcomes.

## Notes

Supplement 1.
Trial Protocol and Statistical Analysis Plan

Supplement 2. **eTable 1.** MVP-ROAR Study Enrollees Excluded From Per-Protocol Analyses or With Otherwise Atypical Results **eTable 2.** Per-Protocol Analyses of 6-Month LDL Cholesterol and Lipid-Lowering Therapy Outcomes Among MVP-ROAR Study Trial Participants **eTable 3.** Changes in Lipid-Lowering Therapy During 6-Month Observation Period Among MVP-ROAR Study Trial Participants **eTable 4.** Participant Responses to Receiving Genetic Results at 6 Months **eTable 5.** Baseline and 6-Month Patient-Reported Outcomes Among MVP-ROAR Study Participants **eTable 6.** Stages of Health Behavior Change Among MVP-ROAR Study Participants at 6 Months

Supplement 3.
Nonauthor Collaborators

Supplement 4.
Data Sharing Statement
