# Supplementary material for: Opportunistic Genomic Screening for Familial Hypercholesterolemia to Improve Low-Density Lipoprotein Cholesterol: A Randomized Clinical Trial
Source: JAMA Netw Open. 2026 Jan 9;9(1):e2549664. doi: 10.1001/jamanetworkopen.2025.49664 (PMC12789956; doi:10.1001/jamanetworkopen.2025.49664)
Supplement: Supplement 1. — Trial Protocol and Statistical Analysis Plan [file jamanetwopen-e2549664-s001.pdf]

Million Veteran Program Return Of Actionable Results – Familial Hypercholesterolemia (MVP-ROAR-FH)

Funding Agency: VA Office of Research and Development

Principal Investigator: Jason L. Vassy, MD, MPH, SM

Version Number 5

March 22, 2022

VA Central IRB
Effective Date: April 26, 2022

Abstract

Background

In the last 5 years, the genomics research and clinical communities have developed some consensus about which disease-associated genetic results are “actionable” for patients and their healthcare providers. At the same time, professional variant interpretation standards and increased data sharing have increased the validity of genetic variant interpretation. Familial hypercholesterolemia (FH) is an example of an actionable monogenic disease, and validated FH variants are now being identified among participants of the Million Veteran Program (MVP). However, it remains uncertain whether patients and their healthcare providers will use such genetic results to change clinical management.

Objectives

The purpose of the MVP-ROAR-FH (Return Of Actionable Results) study is to develop a process to return medically actionable genetic results to living MVP participants and to determine the impact of doing so on medical management and outcomes and Veteran quality of life.

Methods

This a randomized controlled trial of immediate vs. delayed (after 6 months) return of FH variant results. After first being given the opportunity to opt out of participating from the MVP Core study team, living MVP participants with an actionable FH variant are contacted by the MVP-ROAR-FH genetic counselor (GC). The GC discusses the fact that MVP researchers have identified a genetic result that might give the participant and his/her healthcare providers information about his/her risk of heart disease from cholesterol but that this is a research result and would have to be confirmed in a clinical laboratory. After consenting to participate in the study, the participant completes a baseline survey and presents to his/her local VA facility for a blood draw or provides a saliva sample using a self-collection kit. After confirmed biospecimen collection by study staff, participants are randomly allocated to the Immediate Results or Delayed Results arm. Baseline cholesterol values from all participants are measured from blood specimens or, in the absence of a blood draw, are obtained from the medical record. Biospecimens (blood or saliva) from participants in the Immediate Results arm are sent for FH variant confirmation. Upon receiving the variant confirmation results, the GC calls participants in the Immediate Results arm to deliver the study intervention at baseline. Briefly, the GC lets the participant know the results of the FH variant confirmation and delivers standard post-test genetic counseling, including the provision of FH-related resources and other information and facilitation of cascade genetic testing of family members, if appropriate. The GC also sends the results and physician-level materials about FH to the participant’s primary care provider (PCP). Participants in the Delayed Results group receive only their cholesterol results at baseline. During the 6 months after enrollment, participants in both arms continue receiving usual care from their PCPs and other healthcare providers as usual. After 6 months, participants in both arms complete a follow-up survey and undergo an end of study blood draw for repeat cholesterol panel testing and, for participants in the Delayed Results arm, FH variant confirmation testing using either blood or saliva. After their end-of-study data collection is complete, the GC contacts participants in the Delayed Results arm to deliver the same study intervention the Immediate Results arm received. The primary hypothesis is that the reduction in low-density lipoprotein cholesterol after 6 months will be greater in the Immediate Results arm compared to the Delayed Results arm.

VA Central IRB
Effective Date: April 26, 2022

Secondary outcomes include changes in medications, cascade genetic testing among family members, quality of life, and healthcare costs.

Anticipated Impact on Veteran Healthcare

This project has the potential to improve the health care and health outcomes for MVP participants with FH variants while also generating generalizable knowledge about the processes and outcomes of returning genetic results to research participants. The processes developed and studied in this project could inform best practices for the return of genetic results in MVP participants in the VA for other conditions.

VA Central IRB
Effective Date: April 26, 2022

List of Abbreviations

ACC	American College of Cardiology
ACMG	American College of Medical Genetics and Genomics
AE	Adverse event
AHA	American Heart Association
AMP	Association of Molecular Pathology
APOB	*Apolipoprotein B* gene
B	Benign
CHD	Coronary heart disease
CLIA	Clinical Laboratory Improvement Amendments
CVD	Cardiovascular disease
FDA	Food & Drug Administration
FH	Familial hypercholesterolemia
GC	Genetic counselor
GMS	Genomic Medicine Service
HERC	Health Economics Resource Center
III	Individually identifiable information
LB	Likely benign
LDL-C	Low-density lipoprotein cholesterol
LDLR	*Low-density lipoprotein receptor* gene
LP	Likely pathogenic
MCA	Managerial Cost Accounting
MOU	Memorandum of Understanding
MVP	Million Veteran Program
P	Pathogenic
PCP	Primary care provider
PCSK9	*Proprotein convertase subtilisin/kexin type 9* gene
PHI	Protected health information
RCT	Randomized controlled trial
ROAR	Return of actionable results
ROR	Return-of-results
SAE	Serious adverse event
SLCO1B1	*Solute carrier organic anion transporter family member 1B1* gene
SOP	Standard operating procedure
UP	Unanticipated problem
VA	Veterans Affairs
VUS	Variant of uncertain significance

VA Central IRB
Effective Date: April 26, 2022

Table of Contents

1.0 Study Personnel 7

2.0 Introduction 8

3.0 Objectives 10

4.0 Resources and Personnel 12

5.0 Study Procedures 14

5.1 Study Design 14

5.2. Recruitment Methods 21

5.3 Informed Consent Procedures 22

5.4 Inclusion/Exclusion Criteria 23

5.5 Study Evaluations 23

5.6 Data Analysis 26

5.7 Withdrawal of Subjects 27

6.0 Reporting 27

7.0 Privacy and Confidentiality 29

8.0 Communication Plan 31

9.0 References 32

VA Central IRB
Effective Date: April 26, 2022

Appendices

Living MVP Participants with FH Variants (LDLR)

MVP-ROAR-FH Informed Consent Letter

MVP-ROAR-FH Informational Sheet

MVP-ROAR-FH Informational Sheet (Pilot)

MVP-ROAR-FH GC IC Phone Script

MVP-ROAR-FH Instructions for Saliva Collection

MVP-ROAR-FH Baseline Survey

MVP-ROAR-FH Survey Cover Letter

MVP-ROAR-FH Loss to Follow-Up Letter

MVP-ROAR-FH Baseline Letter to Delayed Results Arm

MVP-ROAR-FH Physician Letter (Delayed Results)

MVP-ROAR-FH 6-Month Survey (Immediate Results version)

MVP-ROAR-FH 6-Month Survey (Delayed Results version)

MVP-ROAR-FH Immediate Results 6-Month Letter

MVP-ROAR-FH Physician Immediate Results 6-Month Letter

Sample Lipid Report

MVP-ROAR-FH GC Result Delivery Process

MVP-ROAR-FH Patient Results Letter (Positive / Negative version)

Clinical Variant Report

MVP-ROAR-FH Family Letter

MVP-ROAR-FH Physician Letter (Immediate Results) (Positive / Negative version)

MVP-ROAR-FH Sample Post-counseling CPRS Note

MVP-ROAR-FH Study Withdrawal Letter

VA Central IRB
Effective Date: April 26, 2022

Protocol Title: Million Veteran Program Return Of Actionable Results (MVP-ROAR-FH)

1.0 Study Personnel

1.1 Principal Investigator:

- Jason Vassy, MD, MPH, SM
  Clinician-Investigator, Section of General Internal Medicine,
  VA Boston Healthcare System
  Assistant Professor of Medicine, Harvard Medical School

1.2 Executive committee for the study:

The executive committee will conduct ongoing scientific and operational review of study activities. This committee includes an interdisciplinary collection of clinicians and researchers with expertise in FH, clinical cardiology, genomic medicine, epidemiology, biostatistics, genetic return-of-results, genetic counseling, and econometrics. This committee will meet virtually biweekly, chaired by Dr. Vassy. Members of the executive committee:

- Themistocles (Tim) L. Assimes, MD, PhD
  Associate Director, Epidemiology Research and Information Center for Genomics,
  VA Palo Alto Health Care System
  Associate Professor of Medicine, Stanford University

- Charles A. Brunette, PhD
  Health Science Specialist
  VA Boston Healthcare System

- Kurt D. Christensen, MPH, PhD
  Instructor of Medicine, Harvard Medical School and Brigham and Women's Hospital

- Morgan Danowski, MS, LCGC
  Genetic Counselor
  VA Boston Healthcare System

- Qin Hui, MS
  Genetic Data Analyst, Rollins School of Public Health, Emory University

- Joshua W. Knowles, MD, PhD
  Chief Research Advisor, FH Foundation
  Assistant Professor of Medicine, Stanford University

- Pradeep Natarajan, MD, MMSc

VA Central IRB
Effective Date: April 26, 2022

Director of Preventive Cardiology at Massachusetts General Hospital
Assistant Professor of Medicine, Harvard Medical School

- Amy Sturm, MS, LCGC
  Professor and Director, Cardiovascular Genomic Counseling, Geisinger Health

- Yan Sun, PhD
  Associate Professor of Epidemiology, Emory University

- Virginia Morrison, MS, LCGC
  Genetic Counselor, Genomic Medicine Service,
  VA Salt Lake City Health Care System

- Peter Wilson W.F. Wilson, MD
  Director of Epidemiology and Genomic Medicine, Atlanta VA Medical Center
  Professor of Medicine, Division of Cardiology, Emory University

1.3 Potential participating sites

Because eligible participants for this study include Million Veteran Program (MVP) participants found to have potentially actionable genetic variants, participants may be drawn from more than 50 VA locations, primarily but not limited to those with high MVP enrollment (see Living MVP Participants with FH Variants (*LDLR*)). Study procedures, including recruitment, consent, delivery of the intervention, and data collection will be conducted centrally by the MVP-ROAR study team. MVP local site investigators will be notified each time a participant is enrolled from their site. Recruitment start-up will be graduated. A non-randomized pilot trial will be conducted among 10 eligible MVP participants from the VA Boston Healthcare System and 1-2 other VA locations before initiation of the larger randomized controlled trial (RCT) across the entire MVP cohort.

2.0 Introduction

2.1 Return of results to research biobank participants

Since 2011, MVP has enrolled participants who receive clinical care in the VA healthcare system to understand role of genetics in health[1]. Participants provide broad consent to use their samples and survey and healthcare data for research and consent to be contacted in the future about additional research opportunities. One of the opportunities afforded by biobanks linked to integrated healthcare systems is the ability to screen for genetic risk factors that, if reported to participants and their healthcare providers, may inform their health care and improve health outcomes. Biobank participants routinely state that they would desire and expect actionable medical findings to be returned for their benefit[2,3]. The ethics and practicalities of such return are complicated, but there is an emerging consensus that biobanks should consider reporting clinically validated results in genes deemed medically actionable[4-8].

2.2 Familial hypercholesterolemia: A medically actionable genetic diagnosis

VA Central IRB
Effective Date: April 26, 2022

A common monogenic condition, familial hypercholesterolemia (FH) is an ideal test case for piloting genetic return of results in MVP. Cardiovascular disease, including coronary heart disease (CHD) is the leading cause of death in Veterans. Elevated low-density lipoprotein cholesterol (LDL-C) level is an important, prevalent, and modifiable CHD risk factor[9]. Characterized by extreme LDL-C elevation, FH occurs in approximately 1 in 250 in the US[10-12] and markedly raises premature CHD risk, independent of cross-sectional LDL-C values[10,13,14]. Familial hypercholesterolemia can be caused by variants in the *low-density lipoprotein receptor* (*LDLR*), *apolipoprotein B* (*APOB*), *proprotein convertase subtilisin/kexin type 9* (*PCSK9*) and *low-density lipoprotein receptor adaptor protein 1* (*LDLRAP1)* genes. In current practice, FH may be identified through routine lipid testing, but only 1 in 50 individuals with severe hypercholesterolemia have FH mutations and some FH mutation carriers may not have severely elevated LDL-C. However, across LDL-C values, even when not markedly elevated, FH mutation status portends increased risk of premature CHD. Cholesterol values seen in common hypercholesterolemia can overlap considerably with those seen in FH, particularly in middle-aged and older adults[15,16]. As a result, an estimated 90% of FH cases in the US remain undiagnosed[17].

Compared to common hypercholesterolemia, an FH diagnosis changes prognosis and management, as FH individuals have much greater CHD risk than would be predicted by usual risk models and require earlier and more aggressive therapy and surveillance. Individuals with FH have a 10 to 20-fold higher lifetime CHD risk than those without. Even compared to individuals with equivalent LDL-C levels at a single measurement, FH heterozygotes have a 3-fold higher of CHD compared to matched patients without an FH variant[14,18,19]. Contemporary observational analyses indicate a greater relative and absolute clinical benefit of LDL-C-lowering with statins among those with FH mutations[20]. Thus, the American Heart Association and American College of Cardiology recommend intensive LDL-C lowering (<100 mg/dL or <70 mg/dL, depending on other risk factors) when FH is present, which might necessitate statin dose escalation or the addition of ezetimibe or a PCSK9 inhibitor to high-dose statin therapy[21,22].

Diagnosing FH among MVP participants and distinguishing it from common hypercholesterolemia thus has the potential to improve the healthcare of the Veteran. Considerable data suggest that many patients with FH are undiagnosed and undertreated, resulting in many potentially preventable myocardial infarctions and deaths[23-28]. Indeed, in preparatory-to-research analyses for this proposal, only 240/642 (37%) MVP participants with a potential FH variant in one of the 3 FH genes have a most recent LDL-C <100mg/dL, and only 397/642 (62%) have been prescribed a statin of any dose in the last year. This is consistent with observations in other healthcare settings[29,30]. Moreover, some healthcare providers remain skeptical that a genetic diagnosis of FH should change clinical management over and above non-genetic approaches to risk prediction and management.

Identifying an FH variant in an individual also carries family implications. National registry projects demonstrated the benefits of systematic FH case-finding with genetic screening and cascade testing in family members and show that such an approach may lead to more complete capture, earlier treatment of undiagnosed FH, and cost-effective CHD risk reduction[15,17,31-34]. Thus, several professional organizations and the Centers for Disease Control and Prevention endorse cascade screening among first-degree relatives of patients with an FH variant as an effective genomic medicine intervention with high evidence of clinical utility[17,21]. Because of its actionability, FH is on the American College of Medical Genetics and Genomics (ACMG) list of reportable monogenic conditions[35]. Thus, identification of MVP participants has the potential to improve the lives not only of the Veteran but also their children, siblings and other family members.

VA Central IRB
Effective Date: April 26, 2022

2.3 Emerging consensus around FH variant interpretation and management

Clinical laboratories currently use American College of Medical Genetics and Genomics (ACMG) - Association for Molecular Pathology (AMP) standards and guidelines to classify individual genetic variants into one of five pathogenicity categories for a given disease: benign (B), likely benign (LB), variant of uncertain significance (VUS), likely pathogenic (LP), or pathogenic (P)[36]. In clinical testing, it is standard of care to report only P and LP variants back to ordering providers and patients when unexpected actionable genetic variants are identified[35,37]. The ACMG-AMP standards ask laboratories to apply a set of 28 criteria to classify each variant, using evidence such as population data, computational data, functional data, and segregation data. Once a laboratory interprets a given variant, it is encouraged to share that interpretation in a publicly accessible database such as ClinVar[38], along with any criteria it used to make its determination of pathogenicity. Because there is some subjectivity in how laboratories apply the ACMG-AMP criteria[39], some variants have conflicting interpretations in ClinVar. ClinVar uses a 4-star rating system for the variant interpretations in its database, corresponding to the level of evidence behind each variant classification. Recognizing that content expertise is required to adjudicate the application of ACMG-AMP criteria to specific disease-gene associations, the NIH-funded Clinical Genome Resource (ClinGen) was created in part to implement evidence-based disease expert consensus for curating genes and variants[40]. ClinGen organizes expert curation groups for individual diseases, such as FH. These expert curation groups develop consensus for applying ACMG-AMP criteria in classifying variant pathogenicity for specific diseases and their associated genes. Only variants that are classified by such expert panels can achieve a 3- or 4-star interpretation in ClinVar.

This project will use ClinVar data and the ongoing curation work of the ClinGen Cardiovascular Familial Hypercholesterolemia Variant Curation Expert Panel[41,42] (co-led by Dr. Knowles) to ensure that only variants with high-quality LP or P classifications are returned to participants. As of May 2019, the panel's FH-specific variant criteria have been finalized and are awaiting ClinGen approval, after which the panel will begin to systematically review the >2000 *LDLR* variants listed in ClinVar. We will only consider returning FH variants classified as LP or P according to ACMG-AMP for this project. Thus, it is important to note that, although these are research-derived results, their interpretation will meet clinical standards. Indeed, in December 2018 the US Food and Drug Administration (FDA) formally recognized genetic variant information from the ClinGen Expert Curated Human Genetic Database as a source of valid scientific evidence that can be used to support clinical validity in premarket submissions for diagnostics tests, the first database to receive such recognition.

Although FH may go undiagnosed in routine care, the risk of hypercholesterolemia for CHD in general is widely appreciated in the medical community and among patients. As a result, gaps in genetic literacy may pose less of a barrier for FH return-of-results versus other monogenic conditions. Professional FH guidelines and educational resources exist to help patients and providers manage an FH diagnosis, and these resources will be used in this project to support the responsible and clinically meaningful return of genetic results to MVP participants.

For all the reasons above, FH is the ideal test case for piloting genetic return-of-results in MVP. Although the ACMG lists FH among its actionable conditions and there is emerging expert consensus about how to interpret and manage FH variants, it is unknown whether participants in a biobank study like MVP and their healthcare providers will be receptive to the information and change clinical management accordingly. Moreover, the return-of-results process may bring unanticipated harms, such as distress or anxiety, that would be important to quantify and

VA Central IRB
Effective Date: April 26, 2022

address. Thus, there is equipoise in whether actionable genetic results should be returned to biobank participants. This study will use a randomized design to test the hypothesis that returning FH variants to MVP participants and their providers will result in lower LDL cholesterol levels. If the return-of-results process described in this protocol does not change clinical management or results in undue participant distress, further study will be needed to improve the process and achieve net benefit to patient-participants.

3.0 Objectives

3.1 Study purpose/aims

The purpose of this study is to develop a process to return medically actionable genetic results to living MVP participants and determine the impact of doing so on medical outcomes, Veteran quality of life, and healthcare costs. We will determine this impact by using an RCT of reporting Immediate Results vs. Delayed Results (after 6 months) to test the hypotheses in Section 3.2.

3.2 Study outcomes and hypotheses

3.2.1. Primary outcome

The primary outcome will be the change in LDL-C from study baseline to the end of study (6 months after enrollment). We will test the hypothesis that the LDL-C reduction after 6 months will be greater in the Immediate Results arm compared to the Delayed Results arm.

3.2.2. Secondary outcomes

We will test the following secondary hypotheses:

1. The proportion of participants meeting clinically significant LDL-C targets (< 100mg/dL for primary prevention and <70 mg/dL for secondary prevention) at 6 months will be greater in the Immediate Results arm than in the Delayed Results arm.
2. The proportion of participants with an intensification of lipid-lowering pharmacotherapy will be greater in the Immediate Results arm than in the Delayed Results arm. This composite outcome will include prescription of new monotherapy, dose escalation of existing pharmacotherapy, and addition of one or more medications to existing pharmacotherapy.

3.2.3. Exploratory outcomes

We will test the following exploratory hypotheses:

1. Medication adherence at 6 months will be higher in the Immediate Results arm than in the Delayed Results arm.
2. Participants in the Immediate Results arm will report a greater number of first-degree relatives having undergone genetic testing at 6 months than in the Delayed Results arm.
3. A greater proportion of participants in the Immediate Arm will have healthy lifestyle behaviors (smoking, physical activity, and saturated fat intake) at 6 months than in the Delayed Results arm.

3.2.4. Economic outcomes

VA Central IRB
Effective Date: April 26, 2022

We will perform a budget impact analysis of the intervention alongside the randomized trial, as described in Section 5.6 Data Analysis below.

3.3 Relevance to Veterans and VA

This project has the potential to improve the health care and health outcomes for MVP participants with FH variants while also generating generalizable knowledge about the processes and outcomes of returning genetic results to research participants.

The reporting of potentially pathogenic FH variants to MVP participants and their healthcare providers might impact participants' clinical outcomes in the following ways. First, for Veterans without prior cholesterol testing, receipt of such a variant could prompt them and their providers to initiate routinely recommended healthcare screening. Given the high rates of cholesterol screening in the VA, a small proportion of participants are likely to fall into this category[43]. Second, for Veterans with prior elevated cholesterol results not already on therapy, disclosure of an FH variant result might prompt initiation of statin therapy after discussion with their healthcare provider. Third, an FH variant result will identify participants who are already on lipid-lowering therapy but are undertreated for their level of CHD risk per current guidelines. These participants and their providers might intensify current lipid-lowering regimens, by maximizing statin intensity (medication and dose) or adding additional agents such as ezetimibe or a PCSK9 inhibitor as necessary. The end result for all of these pathways would be an overall reduction in LDL-C values among participants receiving FH variant results, mediated by a change in therapy. Since LDL-C is a well-recognized causal mediator of CHD, LDL-C reduction is an established surrogate endpoint for CHD risk reduction. Beyond the MVP participants themselves, an important additional benefit of reporting FH variants may be a greater uptake of family-based screening among these Veterans' family members.

Moreover, the processes developed and studied in this project could inform best practices for the return of genetic results in MVP participants in the VA for other conditions.

4.0 Resources and Personnel

4.1 Study personnel

All study personnel listed below will have access to protected health information.

4.1.1 Principal investigator

The principal investigator (PI) supervises all aspects of the study. The PI takes ultimate responsibility for the conduct of the study, including meeting study goals, monitoring of participant safety, and dissemination of findings.

4.1.2 Project manager

The project manager (PM) oversees the day-to-day operations of the study, under the supervision of the PI. The PM, in conjunction with the PI and relevant study personnel, develops and maintains study-related standard operating procedures (SOPs). The PM is the primary point of contact between the study personnel and the IRB and works with the IRB to maintain approval for the study protocol and associated documents, including relevant correspondence for protocol modifications, continuing reviews, regulatory

VA Central IRB
Effective Date: April 26, 2022

audits, and event reporting. The PM coordinates and leads regular meetings among the study personnel and collaborators, including preparing meeting agendas and minutes. The PM oversees the study budget and is the primary point of contact between the study and vendors. The PM oversees records management for all study-related documents and materials. The PM may delegate some of these tasks to the research assistant as appropriate. The PM escalates study-related problems to the PI, including any participant safety concerns identified.

4.1.3 Genetic counselor

The genetic counselor (GC) designs and delivers the study intervention to participants and their PCPs. The GC works with the PI and PM to develop the genetic counseling-related materials for review by the IRB. The GC works with the PM to develop the SOP for each step in the return-of-results process, including the ordering of variant confirmation testing, sample acquisition and shipping for variant confirmation testing, receipt of variant confirmation results, reporting of results and associated supportive information to participants and their PCPs, and entry of genetic results into the participants' medical record. The GC describes the study to participants and obtains informed consent. The GC may administer the baseline survey, if conducted at the same time as informed consent. The GC delivers the post-test genetic counseling intervention and works with participants to facilitate cascade genetic testing of family members, as appropriate. The GC is available for follow-up questions by participants and their healthcare providers. The GC is the primary point of contact between the study and the VA-approved, CLIA-certified laboratory used for FH variant confirmation. The GC escalates clinical questions/concerns to the PI, including any participant safety concerns, and escalates research-related questions to the PM.

4.1.4 Senior genetic counselor

The senior GC is a practicing genetic counselor in the VA healthcare system. As applicable, the senior GC guides the use of existing clinical genomic medicine services to facilitate the clinical variant confirmation testing and reporting.

4.1.5. Research assistant

The research assistant (RA) performs study tasks under the supervision of the PM. These include literature reviews; drafting and formatting of IRB and other regulatory documents; preparation of materials for study meetings; formatting of data tables and figures; and drafting of research posters and presentations. The RA performs initial participant eligibility screening by chart review, escalating any questions to the GC. Under the direction of the PM, the RA sends participant mailings at appropriate times. In collaboration with the GC, the RA communicates with local sites to make arrangements for participant blood draws, to facilitate entry of genetic results into the medical record, and to prepare letters and accompanying materials for patients, their families, and PCPs. The RA administers the baseline and end-of-study participant surveys. The RA facilitates the distribution of participation incentives to participants. The RA escalates clinical questions to the GC and research questions to the PM.

4.1.6. Statistician

VA Central IRB
Effective Date: April 26, 2022

The statistician advises the study team on the appropriate study design and statistical analyses for the study outcomes. The statistician conducts sample size and power calculations and supervises the data analyst in performing statistical analysis of study data.

4.1.7 Data analyst

The data analyst (DA) creates and maintains the database housing the study data. The DA creates a data capture system to collect and merge data from participant surveys, medical records, and other VA databases. The DA ensures that data capture and management systems comply with required data security standards. The DA prepares summary data tables for study planning, reporting, monitoring, and dissemination of results. Under the direction of the statistician, the DA performs statistical analyses of study outcomes.

4.2 Services

The study staff will partner with VA Genomic Medicine Service (GMS) to facilitate clinical genetic confirmation testing and report processing for the MVP-ROAR-FH Study. The GMS will provide services and support for FH variant confirmation testing as arranged for MVP-ROAR-FH participants by study staff. GMS uses the VA's telehealth infrastructure to provide clinical genetic services to Veterans in over 90 facilities throughout the country. Part of their service includes an ability to order germline genetic tests for Veterans, with an established process to facilitate testing with commercial laboratories. As applicable, study staff will use established GMS processes to carry out clinical confirmation testing with a VA-approved CLIA-certified laboratory. Biospecimen samples will be labeled with participant name, date of birth, gender, and relevant variant for clinical confirmation, and shipped to the VA Boston Healthcare System clinical laboratory for lipid testing and dissemination to the VA-approved CLIA-certified laboratory for genetic confirmation testing.

5.0 Study Procedures

5.0.1 Study timeline

	**Year 1**				**Year 2**				**Year 3**	
	**Q1**	**Q2**	**Q3**	**Q4**	**Q 1**	**Q2**	**Q3**	**Q4**	**Q1**	**Q2**
**Non-randomized pilot testing of study intervention** (10 eligible MVP participants)										
**Patient recruitment and enrollment** (20-21 patients per month x 12 months = 244)										
**Return of results to patients in immediate results arm**										
**Return of results to patients in delayed results arm**										
**Patient data collection** (6-month outcomes; EHR and survey data)										
**Data preparation and analysis for clinical outcomes**										
**Report on primary outcomes to ORD**										

VA Central IRB
Effective Date: April 26, 2022

**Data preparation and analysis for economic outcomes**										
**Participant and provider qualitative interviews and analysis**										
**Dissemination of results**										

5.1 Study Design

5.1.1. Study overview

This project is an RCT of immediate versus delayed (after 6 months) reporting of clinically confirmed FH variants among 244 MVP participants. In an initial non-randomized pilot phase of the project, the below procedures will be piloted among 10 eligible participants, all of whom will receive their results immediately at baseline.

Figure 1 illustrates the study design, which is further described in the relevant sections below. In brief, the study procedures include the following:

- Living MVP participants with an eligible FH variant are mailed a letter from the MVP Core study team, introducing this new MVP-related study and giving participants the opportunity to opt out of further contact about the study by returning a prepaid opt out postcard or by calling the MVP Call Center.
- To any participant who does not opt out within 2 weeks of this initial mailing, the MVP-ROAR-FH study team mails a letter giving more detail about the study, including all necessary informed consent information (see MVP-ROAR-FH Informed Consent Letter and MVP-ROAR-FH Informational Sheet). Participants in the non-randomized pilot of the study procedures will be mailed a letter introducing the study as well as an informational document including all elements of informed consent (see MVP-ROAR-FH Informed Consent Letter and MVP-ROAR-FH Informational Sheet (Pilot)).
- Two weeks after this mailing, the study genetic counselor calls the participant to review the informed consent information, answers any questions about the study, and documents verbal consent or decline (see MVP-ROAR-FH GC IC Phone Script). Study staff also conduct the baseline survey (see MVP-ROAR-FH Baseline Survey) on this phone call or on another call scheduled at a convenient time for the participant. Participants may complete the baseline survey in paper form by request (see MVP-ROAR-FH Survey Cover Letter). Potentially eligible participants not reached after at least three contact attempts by phone will be sent a loss to follow-up letter (MVP-ROAR-FH Loss to Follow-Up Letter) via certified mail.
- The genetic counselor works with each consented participant and local laboratory staff to facilitate the collection of biospecimen(s). Two tubes of blood will be collected at baseline, one for baseline lipid panel testing and a second for potential CLIA-certified FH variant confirmation (performed by a commercial genetic laboratory). Alternatively, a saliva sample may be collected remotely from the participant for FH variant confirmation using a self-collection kit. Participants may utilize this option if they have undergone lipid testing at their local VA facility within the last 6 months and without a subsequent change in lipid-lowering medications.

VA Central IRB
Effective Date: April 26, 2022

- After the MVP-ROAR-FH study staff confirms baseline biospecimen collection, participants in the RCT are randomly assigned to the Immediate Results arm or the Delayed Results arm. (Participants in the non-randomized pilot will receive their results immediately at baseline).

- Immediate Results arm: The participant's baseline specimen for variant confirmation is shipped to the reference laboratory for clinical variant confirmation of their research results. The laboratory returns these results to the genetic counselor, who schedules a telephone or video visit with the participant, to deliver the intervention described in Section 5.1.3 below. Briefly, he/she lets the participant know the results of the FH variant confirmation and delivers standard post-test genetic counseling, including the provision of FH-related resources and other information. This genetic counseling session is neither audio- nor video-recorded. If the Veteran has living family members, he/she will also educate and provide resources for cascade genetic testing of those family members. The genetic counselor also sends the genetic and cholesterol results and physician-level materials about FH to the participant's PCP. Participants not reached after at least three contact attempts by phone will be sent a loss to follow-up letter (MVP-ROAR-FH Loss to Follow-Up Letter_Result) via certified mail.
    - Delayed Results arm: The participant's baseline blood specimen for variant confirmation is discarded. If collected using saliva, the participant's baseline specimen will be stored

VA Central IRB
Effective Date: April 26, 2022

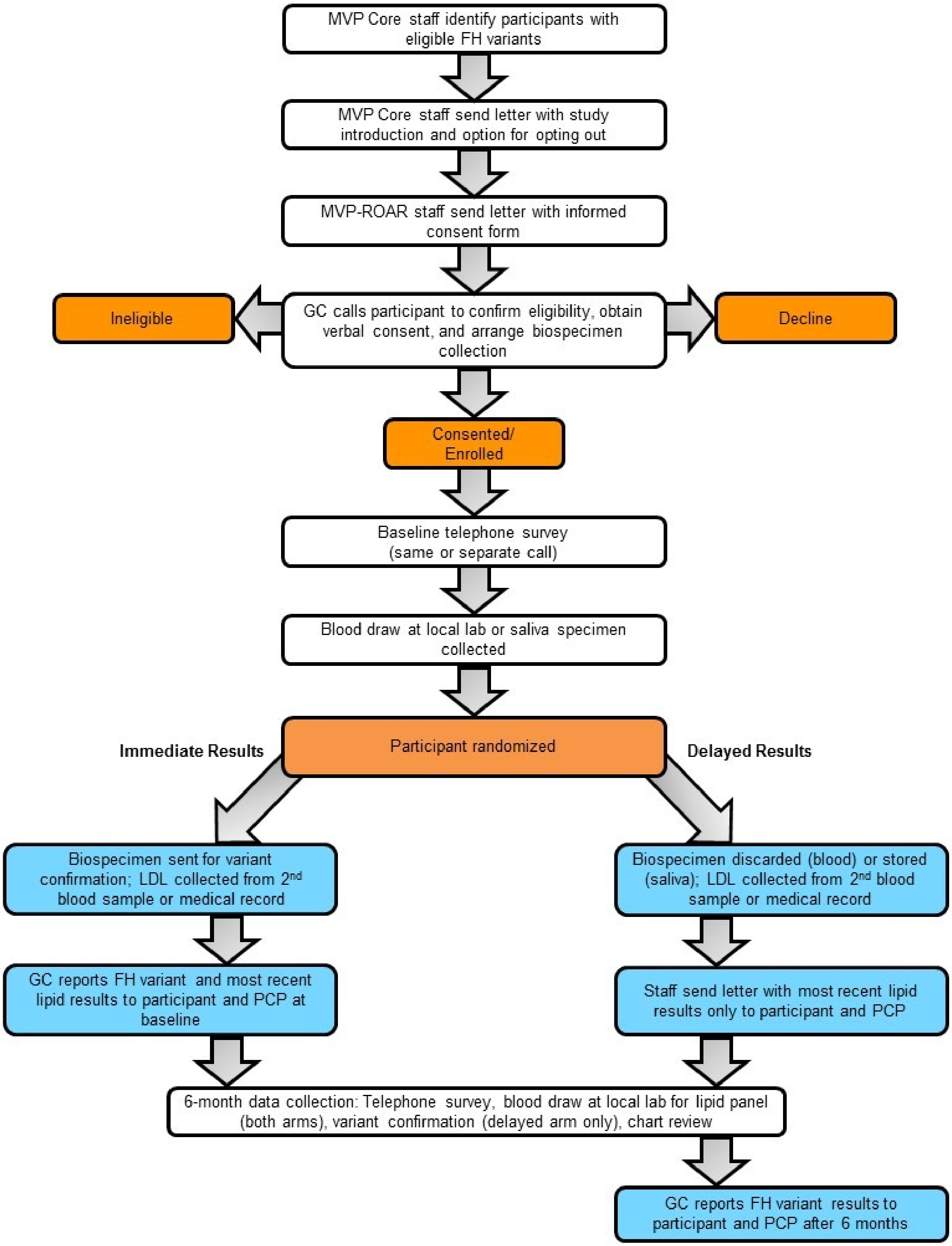

Figure 1: MVP-ROAR study design. Abbreviations: BL, baseline; FH, familial hypercholesterolemia; GC, genetic counselor; MVP, Million Veteran Program; PCP, primary care provider; ROAR, Return Of Actionable Results

VA Central IRB
Effective Date: April 26, 2022

in a locked and secure cabinet for six months, at which time the sample may be shipped to the laboratory for variant confirmation The study staff sends the participant a letter letting them know that they were assigned to the delayed results group and that they will be contacted in 6 months to have their research result confirmed (see MVP-ROAR-FH Baseline Letter to Delayed Results Arm). This letter also includes the participant's baseline cholesterol results. A copy of this letter is also sent to the participant's PCP (see MVP-ROAR-FH Physician Letter (Delayed Results)).

- During the 6 months after enrollment, participants will continue receiving usual care from their PCPs and other healthcare providers. As discussed below in Section 5.1.3, PCPs of patients in the Immediate Results arm may choose to change the patient's treatment or refer the patient to a specialist.
- Six months after randomization, study staff will contact each participant to conduct an end-of-study telephone survey (see MVP-ROAR-FH 6-Month Survey (Immediate Results version) and MVP-ROAR-FH 6-Month Survey (Delayed Results version)) and arrange for the participant to have end-of-study fasting lipid panel testing at their local laboratory. A second specimen, either blood or saliva, will be collected from participants in the Delayed Results arm for clinical variant confirmation testing. Participants may complete the follow-up survey in paper form by request (see MVP-ROAR-FH Survey Cover Letter).
- After their end-of-study data collection is complete, the genetic counselor contacts participants in the Delayed Results arm to deliver the same study intervention the Immediate Results arm received at baseline. Participants not reached after at least three contact attempts by phone will be sent a loss to follow-up letter (MVP-ROAR-FH Loss to Follow-Up Letter_Result) via certified mail.
- After their end-of-study data collection is complete, participants in the Immediate Results arm receive a letter with their follow-up cholesterol results (see MVP-ROAR-FH Immediate Results 6-Month Letter, MVP-ROAR-FH Physician Immediate Results 6-Month Letter, and MVP-ROAR-FH Sample Lipid Report)

5.1.2. Usual care

In this project, usual care is defined as the current approach to screening and management of hypercholesterolemia across the VA healthcare system. Currently, VA locations already achieve high rates of cholesterol screening in their general patient population[43]. Using guidelines such as the 2014 VA/DoD Clinical Practice Guideline for the Management of Dyslipidemia for Cardiovascular Risk Reduction or the 2018 ACC/AHA Multisociety Guideline on the Management of Blood Cholesterol, VA clinicians use cholesterol results, along with other risk factors including blood pressure, diabetes status, and smoking status, to determine whether a Veteran's CVD risk is high enough to merit consideration of treatment with a statin medication. Without other CVD risk factors, VA guidelines do not recommend treating based on elevated LDL-C levels alone unless they are ≥190 mg/dL. Depending on a Veteran's CVD risk, a VA clinician might recommend dietary modification and/or initiate therapy with a statin, such as atorvastatin, simvastatin, or rosuvastatin. Current guidelines recommend that providers repeat cholesterol testing periodically after initiation of therapy to monitor LDL-C reduction. For patients for whom maximum-dose statin therapy is not tolerated or does not achieve sufficient LDL-C reduction, providers might replace or add other medications such as ezetimibe or a PCSK9 inhibitor.

VA Central IRB
Effective Date: April 26, 2022

As discussed in Section 2.2, the guidelines for cholesterol management differ significantly for individuals with FH, although most of these individuals go clinically unrecognized. FH can be clinically recognized without genetic testing using varying definitions, such as the Simon Broome Register criteria, Dutch Lipid Clinic Network criteria, and Make Early Diagnosis to Prevent Early Deaths criteria[19]. However, clinical definitions of FH rely on data that are not necessarily collected systematically in busy clinical practice, such as a detailed family history of hypercholesterolemia or coronary heart disease and the presence of tendinous xanthomata or arcus cornealis on physical examination[19,20]. Recently, an expert consensus panel recommended that genetic testing for FH variants in the *LDLR*, *APOB*, and *PCSK9* genes be performed for any patient meeting a clinical definition for “probable” or “definite” FH[34], but these recommendations have not been widely adopted. Moreover, not all FH patients have an LDL-C ≥190 mg/dL at a given measurement in time. In a recent analysis of data from the Geisinger Healthcare System, only 55% of patients with an FH variant had a maximum LDL-C ≥190 mg/dL in their medical record. Nonetheless, we know that such individuals have a 3-fold higher of CHD compared to matched patients without an FH variant and would thus be recommended to be treated with lipid-lower therapy to a target LDL-C <100mg/dL. Among 325 living MVP participants with a potentially pathogenic *LDLR* variant, 177 (54%) have a most recent LDL-C > 100 mg/dL.

In this context, usual care in this project includes the following:

- VA clinicians, most commonly PCPs, order periodic cholesterol testing for their patients.
- Based on cholesterol results, plus other risk factors including age, sex, blood pressure, diabetes status, prior CVD, and smoking status, PCPs consider initiating treatment with lifestyle modification with or without pharmacotherapy, including statins or other agents.
- PCPs follow these patients at regular intervals, periodically repeating cholesterol testing to monitor for medication adherence and appropriate LDL-C response. PCPs may escalate therapy (*e.g.* increasing the dose of statin therapy or adding ezetimibe to statin therapy) for patients not meeting their LDL-C goals.
- There is significant variability in patients’ willingness to take statin or other medications.
- Depending on individual and regional practice patterns and availability, PCPs may refer certain patients to a preventive cardiology or lipid clinic, if cholesterol results are particularly abnormal or if there is some other concerning feature relevant to the patient’s CVD risk.
- The astute PCP or specialist may perform a detailed physical examination and family history relevant to FH and apply FH clinical criteria to determine whether the patient has a clinical diagnosis of FH.
- For patients meeting clinical criteria for FH, some providers may consider genetic testing for FH variants to make a specific molecular diagnosis of FH (*e.g.* identifying the specific *LDLR* variant causing their disease). This would typically be performed with pre- and post-test genetic counseling, to discuss test results interpretation and implications for family members.

5.1.3. Intervention

VA Central IRB
Effective Date: April 26, 2022

In contrast to usual care, participants enroll in the MVP-ROAR-FH study with the knowledge that analysis of their MVP research sample potentially included information about their heart disease risk that might be useful for them, their healthcare providers, and their family members.

Participants randomly assigned to the Immediate Results arm receive the following study intervention at baseline:

- Participants receive the results of their FH variant confirmation from the research genetic counselor (GC) via telephone or videoconferencing (see MVP-ROAR-FH GC Result Delivery Process). This genetic counseling session includes a detailed family history assessment and specific information about FH, including management guidelines, information about local specialists who treat patients with FH, recommendations for family members, and facilitation of genetic cascade testing of family members.
- A follow-up mailing or encrypted email to participants, corresponding to the variant confirmation result, reiterates the clinical implications (see MVP-ROAR-FH Patient Results Letter Positive or Negative).This mailing also includes a clinical variant report (see Clinical Variant Report), a family letter (see MVP-ROAR-FH Family Letter) that the participant may choose to share with family members, and available FH patient resources, as applicable.
- The GC also sends the participants' PCPs a clinical variant report along with physician-level information about FH treatment guidelines and a list of specialists in the area who treat patients with FH (see MVP-ROAR-FH Physician Letter (Immediate Results) Positive or Negative), as applicable. This letter provides the contact information for the GC, who is available to answer additional questions from providers.
- The clinical variant report is submitted to the participant's local VA for scanning into the medical record (See Clinical Variant Report). The genetic counselor also enters a clinical note in the local medical record summarizing the result (See Sample Post-counseling CPRS Note).
    - In the event the reference laboratory changes the classification of the result (e.g. likely pathogenic to pathogenic variant) the GC will send the participant a letter explaining the change (MVP-ROAR-FH Reclassification Letter). The updated result will also be shared with the participant' PCP and entered into the medical record.
- The essence of the study intervention is a genetic test result and supporting information about its clinical significance. Participants and providers may act on this information as they see fit. The study intervention does not include a specific treatment regimen or other protocolized management strategy.
- If the participant's VA PCP contacts the GC for additional guidance, the GC can offer to facilitate a consultation between the PCP and a cardiologist associated with the study, who can provide management recommendations to the PCP.

Participants randomly assigned to the Delayed Results arm receive a letter informing them of their randomization status and including their cholesterol results (see MVP-ROAR-FH Baseline Letter to Delayed Results Arm). They and their PCPs receive the above intervention 6 months after enrollment, after completing the end-of-study data collection procedures.

<u>5.1.4. Study population</u>

VA Central IRB
Effective Date: April 26, 2022

The eligible study population includes any living MVP participant with a FH-associated genetic variant categorized as Pathogenic or Likely Pathogenic using ACMG-AMP variant interpretation criteria. Among the first >700,000 MVP participants genotyped, approximately 750 meet these criteria. We will enroll approximately 254 participants (10 for the pilot study and 244 for the randomized trial). Per standard clinical practice, the genetic counselor will facilitate cascade genetic testing among family members of participants found to have a clinically confirmed FH variant, but family members will not be considered research subjects, and no research data will be collected about them.

<u>5.1.4.1 Potentially vulnerable subjects</u>

The MVP-ROAR-FH study population may include some subjects considered vulnerable, including students, economically and/or educationally disadvantaged persons, or patients with debilitating or terminal illness. As a result, all potential participants will be informed during the consent process that participation in the study is entirely voluntary and that a decision to not participate or to withdraw from the study at any time has no bearing on the provision of medical care or the receipt of benefits to which the participant is otherwise entitled. Moreover, all participants will be provided opportunities to ask questions of the study staff as well as to consult others, including their families and/or providers, prior to participation. During the study, all participant primary care providers within VA will be provided information regarding their patients' engagement in the study. The study will not include potentially vulnerable participants who are pregnant, incarcerated, or are unable to adequately understand study procedures and provide verbal consent to participate in the study. If it is suspected by the study staff that a participant meets vulnerable subject criteria during recruitment or enrollment, such information will be reported to the study PI for further assessment, action (*i.e.* study withdrawal), and/or required reporting.

<u>5.1.5. Risks to participants</u>

5.1.5.1. Anticipated risks

A principal risk of genetic testing is a breach of confidentiality, in which sensitive information concerning a patient's genetic risk for disease becomes known. Moreover, the return of a genetic test result may lead to the diagnosis of a genetic condition and placement of such information into the medical record. As a result, there may be a risk that genetic information collected from participants during this study is used for the purposes of discrimination with regard to their health insurance or their job. Such recognition of genetic disease risk and/or diagnosis may affect their future insurance costs and/or coverage, such as denial of health, life, disability, or long-term care coverage. There are state, federal, and VA protections that prevent health insurance companies, group health plans, and most employers from discriminating against participants based on their genetic information.

It is possible that participants may be distressed by learning the results of their genetic test. It is also possible that participants may feel anxious or distressed about being randomized to the 6-month Delayed Results arm. Patient assessments including surveys and blood draws may also involve some risk. Some patients may experience distress or discomfort when answering questions about personal demographic and/or medical issues. Common risks associated with venipuncture include minor discomfort, lightheadedness, infection, or bruising at the site of the blood draw.

VA Central IRB
Effective Date: April 26, 2022

5.1.5.2. Minimization of risks

Prior to participation, all participants will undergo a detailed informed consent process by a genetic counselor (see section 5.3 Informed Consent Procedures). The genetic counselor will explain the risks and benefits of genetic confirmation testing and the risks and benefits of participation in this randomized control trial. The informed consent document will include all required elements of consent for study participation, including an explanation of the purposes of genetic testing, a description of known risks associated with genetic testing, a description of any benefits to the patient or others (e.g., familial cascade testing) that may reasonably be expected from genetic testing, and a statement describing the extent, if any, to which confidentiality of patient medical records, data, and/or samples identifying the patient or their genetic test results will be maintained. At any time, patients may elect not to participate.

High levels of distress during patient survey assessments and blood draws are uncommon and staff will be trained to navigate such occurrences. Clinical blood samples will be collected by trained phlebotomists at patients' local VA sites.

Genetic testing results and related clinical information will be returned to participants and their PCPs, for the purposes of medical follow-up, by a trained GC. This information will be documented in the VA medical record, accessible to VA clinicians and others providing routine and/or specialty (*e.g.* cardiology, lipidology, genetics) care.
Participants and their healthcare providers will have access to a dedicated GC throughout the entirety of the study, who will provide pre- and post-genetic test counseling and as-needed consultation. The GC will serve as a resource for both participants and their providers, and, in addition to genetic counseling, may provide education, make appropriate clinical referrals, and be available for additional support, questions, and concerns as necessary. Participants who are noted to be anxious, either by their genetic test result or randomization to the 6-month Delayed Results arm, will be identified by the GC and cases will be discussed with the principal investigator. The GC and principal investigator will use their best clinical judgment to determine the safest path forward for the participant which could include 1) engaging the participant 's provider 2) referral for additional clinical care (e.g., mental health or specialty care), and/or 3) withdrawal from the study (see section 5.7 Withdrawal of Subjects).

There may be other risks that are currently unknown. We will inform participants if any new information is discovered about the risks of taking part in this study and take steps to mitigate them as needed. Any adverse event or reaction experienced by participants in this study, including those associated with genetic testing and/or survey or clinical assessment, will be reported by study staff to the VA Central IRB (see Section 6.0 Reporting).

5.1.5.3. Benefits

Potential benefits to participants include the acquisition of clinical information important to their current and future medical care. Specifically, awareness of FH risk by participants and their providers may aid in the management of a yet unknown, suspected, or current FH diagnosis or for identifying risk of future disease. This information would allow participants to collaborate with their providers to develop improved treatment, surveillance, and/or prevention options related to their FH risk. Early studies also show benefits of family cascade screening as a mechanism to identify at risk family members after known cases of FH are discovered[34].

Benefits to society include an improved understanding of FH genetic testing and return of FH genetic test results. Very limited data exist regarding the benefits or harms of disclosing FH

VA Central IRB
Effective Date: April 26, 2022

mutations to individuals and their families, especially individuals identified through population-level screening, such as MVP, rather than those who are selected for genetic testing due to their own personal or family history of high cholesterol or FH. Population screening for FH genetic variants is not currently the standard of care. This study will allow us to obtain outcomes to explore how participant and provider knowledge of FH impacts medical care compared with currently accepted standards of care. This information may inform future policy, screening, and management guidance for FH, and for the use of genetic testing and the return of results more generally.

5.1.5.4. Comparison of risks and anticipated benefits to patients and society

The risks associated with genetic testing and the return of FH genetic test results in this study are minimal and not dissimilar to what may occur in routine medical care. The potential benefits for confirming and returning FH genetic test results may lead to enhanced awareness of FH and improved treatment for patients with known FH risk. Without rigorously assessing the value of returning FH genetic test results compared to currently accepted standards, the true benefits are unknown. The risk/benefit ratio for the conduct of this study is favorable to the proposed intervention.

5.2. Recruitment Methods

5.2.1. Identification and recruitment of subjects

MVP Core study staff queries MVP databases for living participants with an eligible FH variant. The MVP Core study team mails eligible participants a letter introducing this new MVP-related study and giving participants the opportunity to opt out of further contact about the study by returning a prepaid opt out postcard or by calling the MVP Call Center. To any participant who does not opt out within 2 weeks of this initial mailing, the MVP-ROAR study team mails a letter giving more detail about the study, including all necessary informed consent information (see MVP-ROAR-FH Informed Consent Letter and MVP-ROAR-FH Informational Sheet). Participants in the non-randomized pilot of study procedures will receive a letter introducing the study and an informational document including all elements of informed consent (see MVP-ROAR-FH Informed Consent Letter and MVP-ROAR-FH Informational Sheet (Pilot)).Two weeks after this mailing, the study genetic counselor calls the participant to review the informed consent information, answers any questions about the study, and documents verbal consent or decline (see MVP-ROAR-FH GC ICF Phone Script).

5.2.2. Participant incentives

Participants will be mailed a check for $50 after completing the end-of-study survey and biospecimen collection.

5.3 Informed Consent Procedures

5.3.1 Remote consent

We are requesting a waiver of HIPAA authorization and waiver of documentation of informed consent to enable remote consent by telephone or videoconferencing for eligible MVP participants nationwide.

VA Central IRB
Effective Date: April 26, 2022

5.3.2 Procedure

The study staff will mail eligible participants a letter including all elements necessary for informed consent (see MVP-ROAR-FH Informed Consent Letter, MVP-ROAR-FH Informational Sheet, MVP-ROAR Informational Sheet (Pilot)). The informed consent letter will describe the study, the study procedure, the risks and benefits of participation in the study, confidentiality, data security, and collection and use of health data. Upon receipt of this letter, participants may contact the study staff to ask questions about the study and to arrange a time for the study GC to follow-up for the formal review of informed consent information. If a participant does not contact the study staff after a period of two weeks, the study GC will call the participant to confirm receipt of the informed consent letter and ask whether the participant would like to consent to study participation. Participants uncertain of study participation during this call will be permitted as much time as needed to review the document and to consider enrollment. If the patient is interested in study participation and demonstrates an understanding of the nature of the study and consent process, the GC will formally review the informed consent information with the participant by phone (see MVP-ROAR-FH GC IC Phone Script). The GC will log participant consent in a data file, which will include the date letters are mailed, the dates of participant phone contact, and the date phone consent is obtained. Upon verification of consent, the study staff will conduct the baseline study survey and arrange for the collection of biospecimen during this call or on another call scheduled at a convenient time for the participant. If a participant cannot be reached after at least three attempts to contact them by phone, they will be considered lost to follow-up. These individuals will be sent a letter, including a brief description of the study and study team contact information in the event they become interested or have questions (see MVP-ROAR-FH Loss to Follow-Up Letter). This letter will be sent via certified mail.

5.3.3 Human subjects protection training

The PI and research staff will maintain up-to-date required human subjects training certificates, including Good Clinical Practice, Privacy and HIPAA Focused Training, VA Privacy and Information Security Awareness and Rules of Behavior, and Research Compliance.

5.4 Inclusion/Exclusion Criteria

A participant is eligible for enrollment in this study if he/she meets the following criteria:

- Is a living enrollee in MVP
- Is identified to have a Pathogenic or Likely Pathogenic variant in an FH-associated gene in their MVP genotype data (see Section 2.3)
- Has not previously undergone genetic testing for familial hypercholesterolemia. Study staff first ascertain this by review of the medical record and then confirm during the informed consent call by asking the participants about any prior genetic testing he/she has undergone (see MVP-ROAR-FH GC IC Phone Script).
- Is not incarcerated
- Is not pregnant

VA Central IRB
Effective Date: April 26, 2022

5.5 Study Evaluations

Study data will come from the following sources and procedures:

5.5.1. MVP database

The MVP Core study staff will provide the MVP Return study staff a data file with the following information about potentially eligible participants:

- Name, date of birth, and last 4 digits of Social Security Number (SSN)
- Genotype in the *APOB*, *LDLR*, *LDLRAP1*, *PCSK9*, and *SLCO1B1* genes from their MVP genetic data
- Race and ethnicity
- VA station where participant enrolled in MVP and VA station(s) where participant currently receives health care

5.5.2. Medical record review

Study staff will review each potentially eligible participant's medical records to confirm the absence of a prior positive genetic test result for FH and to abstract the participant's list of medications.

5.5.3. Informed consent call

At the beginning of the informed consent call (see MVP-ROAR-FH GC IC Phone Script), the GC confirms the participant's name, date of birth, and last 4 SSN digits. He/she then also asks whether the participant has had prior genetic testing and, if so, what kind. Participants whose responses indicate, in the clinical judgement of the GC, a prior positive genetic test result for FH are ineligible. During this call the GC will collect or confirm participant contact information including preferred phone number, mailing address, and an email address.

5.5.4. Baseline telephone survey

The baseline survey (see MVP-ROAR-FH Baseline Survey) may occur on the same phone call as the informed consent process or on a separate call. Participants may complete the baseline survey in paper form by request (see MVP-ROAR-FH Survey Cover Letter). The survey collects the following information from the consented participant:

- Name and station of participant's VA PCP
- Name and contact information of any PCP outside of VA, if applicable
- Confirmation of baseline VA medication list, as assessed from medical record review

VA Central IRB
Effective Date: April 26, 2022

- Medication name and dose for any over-the-counter medications or medications prescribed by a provider outside VA
- Beliefs about medications: Beliefs About Medicines Questionnaire (BMQ)[44]
- Patient activation: Patient Activation Measure (PAM)[45]
- Quality of life: Veterans RAND 12-item Health Survey (VR-12)[46]
- Race and ethnicity

5.5.5. Baseline biospecimen collection

Participants present to their local laboratory for a fasting blood draw for two tests:

- Baseline lipid panel testing: This is performed by a clinical laboratory at a VA facility.
- FH variant confirmation: For participants randomized to the Immediate Results arm, this is performed by an external Clinical Laboratory Improvement Amendments (CLIA)-certified, College of American Pathologists (CAP)-accredited laboratory. This laboratory uses standard clinical techniques to sequence FH-associated genes and confirm the presence or absence of the suspected FH variant from the MVP genetic data. The laboratory sends a typical clinical report back to the study staff (see Clinical Variant Report). For participants in the Delayed Results arm, the second baseline blood specimen will be discarded.

  Participants for whom it is not feasible to visit their local VA facility for a blood draw may provide a self-collected at-home saliva sample for FH variant confirmation. To utilize this option, participants must have:
  - A documented LDL-C level in their VA health record not older than 6 months from their date of enrollment
  - Absence of a statin prescription change within the time from the most recent documented LDL value to enrollment

  For participants in the Immediate Results arm, the saliva sample will be returned to the study staff and shipped to the CLIA-certified laboratory for FH variant confirmation. For participants in the Delayed Results arm, the saliva sample will be returned to the study staff and stored in a locked and secure cabinet for six months, at which time the sample may be shipped to the laboratory for variant confirmation or discarded if the participant is able to visit their local VA facility for a blood draw.

5.5.6. End-of-study telephone survey

Six months after enrollment, the study staff calls each enrolled participant to administer the end-of-study survey (see MVP-ROAR 6-Month Survey (Immediate Results version) and MVP-ROAR-FH 6-Month Survey (Delayed Results version)). Participants may request to complete the follow-up survey by paper (see MVP-ROAR-FH Survey Cover Letter). The follow-up survey includes the following questions and instruments:

- Confirmation of current VA medication list, as assessed from chart review

VA Central IRB
Effective Date: April 26, 2022

- Medication name and dose for any current over-the-counter medications or medications prescribed by a provider outside VA
- Beliefs about medications: Beliefs About Medicines Questionnaire (BMQ)
- Health behaviors: Smoking status[47], saturated fat intake[48], physical activity[49]
- Quality of life: Repeated measurement of VR-12[46]
- Healthcare utilization, including laboratory tests, office visits to PCP and specialists, and hospitalization[50]
- Veteran time demands and transportation costs to attend CHD-related medical appointments[51]
- Whether and how many first-degree family members underwent cascade genetic testing
- Feelings about genomic testing results: End-of-study measurement of FACToR[52]
- Preferences for receiving genetic test results

5.5.7. End-of-study biospecimen collection

After the end-of-study survey, participants present to their local laboratory for an end-of-study fasting blood draw:

- End-of-study lipid panel testing: This is performed by VA clinical laboratory.
- FH variant confirmation: For participants randomized to the Delayed Results arm, a second blood specimen is drawn or self-collected saliva sample obtained for FH variant confirmation at an external reference laboratory, as described in Section 5.5.5 above.

5.5.8. End-of-study VA database review

Study staff will review each enrolled participants' medical record, Corporate Data Warehouse data, and other VA databases for the following clinical processes and outcomes related to FH:

- Lipid panel results prior to enrollment and during the study period
- Other clinical tests or procedures related to CVD, such as coronary computed tomography scan, C-reactive protein, lipoprotein(a), electrocardiogram (ECG), transthoracic echocardiogram, stress test, angiography, coronary artery bypass surgery
- All prescriptions of lipid-lowering medications prior to enrollment and during the study period
- Diagnoses related to hypercholesterolemia, FH, CHD, ischemic stroke, peripheral artery disease
- Healthcare utilization and cost data from the Health Economics Resource Center (HERC) and Managerial Cost Accounting System (MCA) datasets.

5.5.9. Intervention cost accounting

VA Central IRB
Effective Date: April 26, 2022

The costs of the intervention, including materials and personnel time, will be collected to enable the budget impact analysis described in Section 5.6.2.

5.6 Data Analysis

5.6.1. Sample size determination

We will enroll a total of 254 participants: 10 for the non-randomized pilot trial and 244 for the RCT.

Sample size is based on the primary outcome of change in LDL-C in each arm after 6 months. Assuming a mean LDL-C reduction of 20% in the Immediate Results arm, a mean LDL-C reduction of 0% in the Delayed Results arm, and a common standard deviation of 30%[53], 72 total participants (36 per arm) are needed to have 80% power to detect a significant between-group difference at alpha=0.05. Enrollment of twice this number (144 total) will account for an absence of therapy escalation in up to 50% of participants in the Immediate Results arm. Enrollment of 180 total participants will account for up to 20% loss to follow-up.

Change in LDL-C at 6 months			Total sample size required
**Immediate Results**	**Delayed Results**	**Common SD**	
-20%	0%	30%	72
-20%	0%	40%	126
-20%	-5%	30%	126
-20%	-5%	40%	224

An important secondary outcome is the proportion of participants in each arm meeting accepted LDL-C targets at 6 months (<100 mg/dL for primary prevention and <70 mg/dL for secondary prevention). In preparatory-to-research analyses, only 175/322 (46%) MVP participants with a potentially pathogenic *LDLR* variant had a most recent LDL-C <100mg/dL. To have 80% power to detect a between-arm difference of 20% of participants meeting this LDL-C target at alpha=0.05, up to 194 total participants are needed (97 per arm). To account for up to 20% loss to follow-up, a total of 244 participants (122 per arm) are needed.

Proportion of participants with LDL-C < 100mg/dL at 6 months		Total sample size required
**Immediate Results**	**Delayed Results**	
10%	30%	124
20%	40%	162
30%	50%	186
40%	60%	194

VA Central IRB
Effective Date: April 26, 2022

5.6.2. Data analysis plan

We will conduct intention-to-treat analyses to compare all outcomes in the Immediate and Delayed Results arms. Logistic regression will be used for dichotomous outcomes, and linear regression will be used for continuous outcomes. Poisson regression will be used to compare counts of first-degree family members undergoing cascade genetic testing between the two arms. Regression models will test the study hypotheses by including terms for randomization status. Covariates may be included if they improve model precision. Missing data will be imputed using fully conditional specification.

We will perform a budget impact analysis of the intervention alongside the randomized trial, using International Society for Pharmacoeconomics and Outcomes Research[54] and Second Panel on Cost-Effectiveness in Health and Medicine[51] recommendations. We will use administrative data and microcosting and gross costing strategies[55] to estimate the cost of the return-of-results intervention itself plus patient-level healthcare costs extracted from the VA Corporate Data Warehouse from the 6 months after enrollment. Mean Veteran health-related quality of life (VR-12) will be compared between the two study arms and used to inform cost-effectiveness analyses by mapping scores to the VR-6D to estimate health utilities.

All data will be stored on encrypted, password-protected VA servers and analyzed only by credentialed individuals identified as study staff on the IRB protocol.

5.7 Withdrawal of Subjects

Study subjects will be made aware that they may withdraw from participation in this study at any time without penalty or loss of VA or other benefits to which they are entitled. It is expected, given the limited risk of the study intervention, the reporting of relevant information to participants' providers, and participants' receipt of usual care throughout their study participation, that subject withdrawal will be rare. If study staff develop serious concerns that a participant is highly distressed, anxious, depressed, or whose health and well-being may otherwise be immediately compromised as a result of his or her participation in the study, and at the discretion of the PI, the study team may inform the participant that he or she may withdraw from the study. Researchers may continue to use patient data collected prior to withdrawal. No further health data will be collected after a participant has withdrawn from the study.

Study participants may withdraw from the study by contacting the study team by phone and requesting withdrawal verbally. Once a verbal request for withdrawal is received by the study team, participants are provided with confirmation of their withdrawal from the study (see MVP-ROAR-FH Study Withdrawal Letter). If a participant withdraws before the baseline biospecimen collection, no information about the participant's MVP research result will be provided to the participant. If a participant withdraws after the baseline biospecimen collection but before the reference laboratory has analyzed his/her sample, the study staff will contact the lab to destroy the sample before analysis. If a participant withdraws after the laboratory analysis but before receiving his/her results from the GC, he/she will be given the opportunity to receive the results from the GC and/or have them sent to his/her PCP, 6 months after enrollment.

6.0 Reporting

6.1. Monitoring and quality assurance

VA Central IRB
Effective Date: April 26, 2022

The PI will monitor the study proceedings to ensure the study is executed according to the IRB-approved protocol, which includes ensuring the safety of participants and the validity, integrity, and protection of data associated with this study. The PI and research staff will be responsible for the day-to-day monitoring of patient safety and data quality throughout the conduct of this study. In addition to the regular monitoring of patient safety and data quality, the research team will complete any and all continuing reviews, audits, event reporting, and/or other requirements per the provisions and timelines set forth by VA Central IRB.

Any concerns regarding the ethical conduct of the study, the safety of participants, or a breach in the protection of study data made by participants, the study staff, or others will be promptly reported to the PI and escalated accordingly to VA Central IRB and/or other relevant oversight committees.

6.2 Participant safety

Participant safety will be monitored by the PI and study staff throughout the conduct of study activities. All participants will have the contact information of the MVP Information Center and the MVP-ROAR-FH study team for any questions or concerns related to study participation. All participants will also have the contact information of the study GC should they have any concerns with their results or their delayed result disclosure. Per the study protocol the participant’s PCP will also receive a copy of the participant’s genetic test results. If a participant experiences adverse effects or expresses emotional distress related to study participation at any time during their enrollment and requires medical attention based on the judgement of the PI, the study genetic counselor, and/or their PCP, they will be referred for clinical assessment and/or informed of options for study withdrawal (see Section 5.7 Withdrawal of Subjects) as appropriate. All serious such cases, including those requiring a referral to a mental health professional or other therapeutic intervention, will be reviewed and reported to VA Central IRB as an adverse event (AE) or serious adverse event (SAE) as necessary.

Throughout the conduct of the study, attention will be paid to any reports of participant experiences that constitute adverse events as described in Section 6.3 Adverse events and will be reported per Section 6.4 Event reporting.

6.3 Adverse events

Adverse events (AEs) related to MVP-ROAR-FH procedures do not include anticipated events related to blood draws (*e.g.*, pain, minor bleeding, bruising, fainting, or lightheadedness) and minor feelings of discomfort while answering survey questions. Pre-existing conditions or illnesses which are expected to exacerbate or worsen are also not considered adverse events and will be accounted for in the subject’s medical history. An AE may be considered any other unanticipated or unintended medical occurrence or worsening of a sign or symptom (including an abnormal laboratory finding other than the return of genetic information associated with FH) or disease in a study subject, which does not necessarily have a causal relationship with the study condition, procedure(s) or study agent(s), that occurs after participant informed consent is obtained. A serious adverse event (SAE) will be defined as an AE resulting in one of the following outcomes: death during the 6 months after study enrollment, life threatening event (defined as an event that places a participant at immediate risk of death), inpatient hospitalization, and any other condition which, in the judgment of the PI, represents a significant hazard, such as an important medical event that does not result in one of the above outcomes. An event may be considered an SAE when it jeopardizes the participant or requires medical or

VA Central IRB
Effective Date: April 26, 2022

surgical intervention to prevent one of the outcomes listed above. AEs may be observed by the study staff or volunteered by participants, their family members, their PCPs, or others. All AEs and SAEs will be assessed for relationship to the study research procedures by the study PI, to determine whether study participation was likely to have caused the AE/SAE.

6.4 Event reporting

Given the minimal risk of the study intervention, the study team anticipates few AEs, SAEs, or unanticipated problems (UPs) during the course of this study. AEs, SAEs, and UPs may be observed through regular monitoring by the study staff or volunteered by participants, their family members, their PCPs, or others throughout the conduct of the study. Any concerns related to patient safety or the potential occurrence of an AE, SAE, or UP in relation to the conduct of this study will be promptly reported to the PI for review. Upon discovery, any study-related death will be immediately reported to the VA Central IRB. Study staff will report any protocol deviation that substantively affects subjects' rights or safety, UP that poses risk to participants, or SAE per VA Central IRB protocols. An annual report summarizing all non-serious adverse events and UP/protocol deviations that did not require immediate reporting will be prepared and reported to the VA Central IRB at the time of continuing review. Acknowledgement of any AE, SAE, or UP/protocol deviation by the VA Central IRB will be retained by the MVP-ROAR-FH study staff.

7.0 Privacy and Confidentiality

7.1 Use of Protected Health Information (PHI)

The MVP-ROAR-FH study team will utilize participant Protected Health Information (PHI) during the conduct of study activities, including in the identification and recruitment of eligible participants, for genetic confirmation testing, for the delivery of the study intervention, and for the collection of study data. Given the use of PHI, every effort will be made to ensure that the privacy and confidentiality of MVP-ROAR-FH participants are maintained. As with any research study, loss of privacy and/or confidentiality is a potential risk. To mitigate this risk, study personnel will take every precaution to keep participants' PHI confidential and protect each participant's privacy in all aspects in which participant data is used as part of this research study. This includes using coded information rather than PHI whenever possible, storing electronic study-related data on VA and/or VA-approved servers in accordance with appropriate information security policies, using only VA-approved methods for the transfer of data, and storing any participant-related written and/or paper documents in a secure environment (e.g., locked cabinet and in a locked office).

7.1.1 Biospecimens and genetic variant data

Blood specimens will be collected at participants' local VA facilities and shipped to the VA Boston Healthcare System laboratory for FH variant confirmation testing at a VA-approved CLIA-certified laboratory. Saliva collection kits will be sent directly to participants using a common carrier delivery service and will be tracked with a unique reference number. Saliva samples will be self-collected in the participant's home and shipped to study staff for FH variant confirmation testing at a VA-approved CLIA-certified laboratory. All participant data will be retained within the VA except the following in order to complete genetic variant confirmation testing: patient name, date of birth, gender, and a test requisition form will be included with the blood sample sent to the VA-approved CLIA-certified laboratory for genetic confirmation testing,

VA Central IRB
Effective Date: April 26, 2022

using a common carrier delivery service and chain of custody. Specimens will be shipped to the VA-approved clinical laboratory via prepaid standard biospecimen collection kits. The shipment to the VA-approved CLIA-certified laboratory will not include any personal identifiers on the external packaging. Each shipment will be distributed for overnight delivery and will be tracked with a unique reference number. The VA-approved CLIA-certified laboratory will perform variant confirmation and then retain the sample only as long as is required by internal quality assurance and other regulatory procedures. The VA-approved CLIA-certified laboratory will return the results of variant confirmation testing to the study staff via a secure, password protected, and VA-approved web-interface. The study staff will transfer the clinical variant confirmation test results to the medical record at participants' local VA facilities. Copies of the participants' results will also be provided to their designated VA PCPs via secure VA-approved methods.

7.2 Staff training and data access

All study staff with access to PHI will complete required training and be instructed, in accordance with VA policy, on the requirements of Federal privacy and information laws and regulations, VA regulations and policies, and VHA policy. Only study personnel credentialed and approved by the VA Central IRB and VA Research & Development committees will have access to study data stored in either physical or electronic environments. Once study team members are no longer a part of the research team, their access to data and research materials will be terminated. No unauthorized access to study servers or datasets will be permitted. All study staff will be trained on reporting data breaches within one (1) hour to the appropriate Information Security and Privacy Officers and VA Central IRB. Access to all electronic data and files (e.g., database, spreadsheet) containing identifiable patient information will be limited to approved users with a login credential. Any computer hosting such files will be password protected to prevent access by unauthorized users.

7.3 Data security

Risk of breach of confidentiality will be minimized through the appropriate management and security of participant data per VA, HIPAA, and MVP requirements. Participant PHI will be delinked from the final analytic dataset. All data will be retained within the VA. Data will be securely transmitted using VA approved methods, including FIPS 140-2 validated encryption. This will include transmission of PHI and other participant data, including genetic testing results, between VA and the commercial reference laboratory performing genetic confirmation testing. Participant data files (source and analytic) will be stored behind the VA firewall, on a drive created specifically to house the data for this research project. No PHI (including scrambled SSNs or dates) will be released to the public, nor will they be presented or published in the dissemination of study findings. All written and/or paper documents containing participant PHI will be filed in a locked cabinet and locked office at the VA Boston Healthcare System. Study data will be kept indefinitely or until the law allows their destruction in accordance with the VA Record Control Schedule. Electronic records will be destroyed, when allowed, in a manner in which they cannot be retrieved. In addition, a Certificate of Confidentiality, issued by the National Institutes of Health (NIH), will help to ensure the privacy of participant identities and data. With this certificate, researchers cannot be forced (e.g. by court subpoena) to disclose information that identify the participant in federal, state, or local civil, criminal, administrative, legal or other proceedings.

7.3.1 Participant mailing data

VA Central IRB
Effective Date: April 26, 2022

A copy of participant mailing data only will be downloaded outside of VINCI and stored on a secure, study specific SharePoint site. The SharePoint site will be housed behind the VA firewall and viewing of mailing data will be limited to IRB approved study personnel. This will be done to enable the use of Microsoft Word mail merge software to create participant letters and address labels for efficient printing and distribution. Participant mailing data will be in the form of CSV files and may include identifying variables including: participant ID, full name, gender, mailing address, and any associated flags (i.e. temporary address). The mail merge software can be used within the secure SharePoint environment.

7.3.2 Use of encrypted emails

The MVP-ROAR-FH Study will include the option for participants and their providers to receive encrypted email correspondence associated with IRB-approved study materials and study-related appointments (*i.e.,* local facility blood draws, remote genetic counseling appointments). Per VA policy, any email distributed external to the VA network and including individually identifiable information (III), PHI, or study specific information will be encrypted using the VA-approved Microsoft Azure Rights Management Service (RMS).

Only after initiating contact by standard mail and telephone, and after participants have had the opportunity to opt out of contact, participants will have the option to request that study staff send recruitment and other study materials (i.e. informational sheet, baseline survey, end-of-study survey, delayed arm letter, VA form 10-5345, results mailings) which may contain PHI or study-specific information via Azure RMS encrypted email. Participants may also opt to return study materials (i.e. baseline survey, end of study survey, and VA form 10-5345) by scanning them or taking a photo and returning it to study staff via Azure RMS encrypted email.

8.0 Communication Plan

8.1 Notification of local facility

As an MVP-associated study, MVP local site investigators will be made aware of the conduct of MVP-ROAR-FH and that MVP participants from their facilities may be recruited for enrollment into this study. In the event a SAE or unanticipated problem occurs in conjunction with the conduct of MVP-ROAR-FH, the MVP local site investigator of the affected participant will be informed and the event will be reported in accordance with VA Central IRB procedures.

8.2 Public data set

We will share de-identified individual-level trial data (participant characteristics and outcomes) through a data repository housed on a secure VA server and accessible only to outside investigators with IRB and other regulatory approvals. In the event of a data request, approval for data use will be sought from the local IRB where the repository is housed.

8.3 VA MVP Central Database

Identifiable, individual-level trial data will be shared with the MVP Program and will be stored in the VA MVP Central Database.

8.4 Dissemination of study results

VA Central IRB
Effective Date: April 26, 2022

This trial will be registered on ClinicalTrials.gov. Communication of study progress and results will be the responsibility of the PI and Executive Committee. Information associated with this project will be communicated to VA and other stakeholders including policymakers, healthcare providers, patients, and the scientific community. In order to broadly disseminate information about this research, the MVP-ROAR-FH study team 1) will prepare scientific presentations and manuscripts for publication, 2) present information regarding the purpose, methods, and results of this research at informal meetings throughout the VA, 3) inform the public and policymakers of new findings through press releases and related mechanisms, including those currently approved through MVP, and 4) facilitate dissemination of study-related information and findings to patients and healthcare providers through collaboration with MVP leadership, VA national leadership, and other centers.

9.0 References

1. Gaziano JM, Concato J, Brophy M, et al. Million Veteran Program: A mega-biobank to study genetic influences on health and disease. *J Clin Epidemiol.* 2016;70:214-223.
2. Allen NL, Karlson EW, Malspeis S, Lu B, Seidman CE, Lehmann LS. Biobank participants' preferences for disclosure of genetic research results: perspectives from the OurGenes, OurHealth, OurCommunity project. *Mayo Clin Proc.* 2014;89(6):738-746.
3. Kaufman D, Murphy J, Erby L, Hudson K, Scott J. Veterans' attitudes regarding a database for genomic research. *Genet Med.* 2009;11(5):329-337.
4. Bredenoord AL, Kroes HY, Cuppen E, Parker M, van Delden JJ. Disclosure of individual genetic data to research participants: the debate reconsidered. *Trends Genet.* 2011;27(2):41-47.
5. Bredenoord AL, Onland-Moret NC, Van Delden JJ. Feedback of individual genetic results to research participants: in favor of a qualified disclosure policy. *Hum Mutat.* 2011;32(8):861-867.
6. Levesque E, Joly Y, Simard J. Return of research results: general principles and international perspectives. *J Law Med Ethics.* 2011;39(4):583-592.
7. Wolf SM. Return of individual research results and incidental findings: facing the challenges of translational science. *Annual review of genomics and human genetics.* 2013;14:557-577.
8. Knoppers BM, Zawati MH, Senecal K. Return of genetic testing results in the era of whole-genome sequencing. *Nat Rev Genet.* 2015;16(9):553-559.
9. Bibbins-Domingo K, Grossman DC, Curry SJ, et al. Screening for Lipid Disorders in Children and Adolescents: US Preventive Services Task Force Recommendation Statement. *JAMA.* 2016;316(6):625-633.
10. Youngblom E, Pariani M, Knowles JW. Familial Hypercholesterolemia. In: Adam MP, Ardinger HH, Pagon RA, et al., eds. *GeneReviews((R)).* Seattle (WA): University of Washington, Seattle; 1993.
11. Sniderman AD, Tsimikas S, Fazio S. The severe hypercholesterolemia phenotype: clinical diagnosis, management, and emerging therapies. *J Am Coll Cardiol.* 2014;63(19):1935-1947.
12. Dron JS, Hegele RA. Genetics of Lipid and Lipoprotein Disorders and Traits. *Current genetic medicine reports.* 2016;4(3):130-141.
13. Benn M, Watts GF, Tybjærg-Hansen A, Nordestgaard BG. Mutations causative of familial hypercholesterolaemia: screening of 98 098 individuals from the Copenhagen General Population Study estimated a prevalence of 1 in 217. *Eur Heart J.* 2016;37(17):1384-1394.

VA Central IRB
Effective Date: April 26, 2022

14. Khera AV, Won HH, Peloso GM, et al. Diagnostic Yield and Clinical Utility of Sequencing Familial Hypercholesterolemia Genes in Patients With Severe Hypercholesterolemia. *J Am Coll Cardiol.* 2016;67(22):2578-2589.
15. Umans-Eckenhausen MA, Defesche JC, Sijbrands EJ, Scheerder RL, Kastelein JJ. Review of first 5 years of screening for familial hypercholesterolaemia in the Netherlands. *Lancet.* 2001;357(9251):165-168.
16. Jacobson TA, Maki KC, Orringer CE, et al. National Lipid Association Recommendations for Patient-Centered Management of Dyslipidemia: Part 2. *J Clin Lipidol.* 2015;9(6, Supplement):S1-S122.e121.
17. Knowles JW, Rader DJ, Khoury MJ. Cascade Screening for Familial Hypercholesterolemia and the Use of Genetic Testing. *JAMA.* 2017;318(4):381-382.
18. Nordestgaard BG, Chapman MJ, Humphries SE, et al. Familial hypercholesterolaemia is underdiagnosed and undertreated in the general population: guidance for clinicians to prevent coronary heart disease: consensus statement of the European Atherosclerosis Society. *Eur Heart J.* 2013;34(45):3478-3490a.
19. Nanchen D, Gencer B, Muller O, et al. Prognosis of Patients With Familial Hypercholesterolemia After Acute Coronary Syndromes. *Circulation.* 2016;134(10):698-709.
20. Besseling J, Hovingh GK, Huijgen R, Kastelein JJP, Hutten BA. Statins in Familial Hypercholesterolemia. *Consequences for Coronary Artery Disease and All-Cause Mortality.* 2016;68(3):252-260.
21. Gidding SS, Champagne MA, de Ferranti SD, et al. The Agenda for Familial Hypercholesterolemia: A Scientific Statement From the American Heart Association. *Circulation.* 2015;132(22):2167-2192.
22. Grundy SM, Stone NJ, Bailey AL, et al. 2018 AHA/ACC/AACVPR/AAPA/ABC/ACPM/ADA/AGS/APhA/ASPC/NLA/PCNA Guideline on the Management of Blood Cholesterol. *Circulation.*0(0):CIR.0000000000000625.
23. Versmissen J, Oosterveer DM, Yazdanpanah M, et al. Efficacy of statins in familial hypercholesterolaemia: a long term cohort study. *BMJ.* 2008;337:a2423.
24. Do R, Stitziel NO, Won HH, et al. Exome sequencing identifies rare LDLR and APOA5 alleles conferring risk for myocardial infarction. *Nature.* 2015;518(7537):102-106.
25. Neil HA, Hammond T, Huxley R, Matthews DR, Humphries SE. Extent of underdiagnosis of familial hypercholesterolaemia in routine practice: prospective registry study. *BMJ.* 2000;321(7254):148.
26. Benn M, Watts GF, Tybjaerg-Hansen A, Nordestgaard BG. Familial hypercholesterolemia in the danish general population: prevalence, coronary artery disease, and cholesterol-lowering medication. *J Clin Endocrinol Metab.* 2012;97(11):3956-3964.
27. Huijgen R, Vissers MN, Defesche JC, Lansberg PJ, Kastelein JJ, Hutten BA. Familial hypercholesterolemia: current treatment and advances in management. *Expert Rev Cardiovasc Ther.* 2008;6(4):567-581.
28. Mortality in treated heterozygous familial hypercholesterolaemia: implications for clinical management. Scientific Steering Committee on behalf of the Simon Broome Register Group. *Atherosclerosis.* 1999;142(1):105-112.
29. Abul-Husn NS, Manickam K, Jones LK, et al. Genetic identification of familial hypercholesterolemia within a single U.S. health care system. *Science.* 2016;354(6319).
30. Rodriguez F, Knowles JW, Maron DJ, Virani SS, Heidenreich PA. Frequency of Statin Use in Patients With Low-Density Lipoprotein Cholesterol >/=190 mg/dl from the Veterans Affairs Health System. *Am J Cardiol.* 2018;122(5):756-761.

VA Central IRB
Effective Date: April 26, 2022

31. Wonderling D, Umans-Eckenhausen MA, Marks D, Defesche JC, Kastelein JJ, Thorogood M. Cost-effectiveness analysis of the genetic screening program for familial hypercholesterolemia in The Netherlands. *Semin Vasc Med.* 2004;4(1):97-104.
32. Ademi Z, Watts GF, Pang J, et al. Cascade screening based on genetic testing is cost-effective: evidence for the implementation of models of care for familial hypercholesterolemia. *J Clin Lipidol.* 2014;8(4):390-400.
33. Alver M, Palover M, Saar A, et al. Recall by genotype and cascade screening for familial hypercholesterolemia in a population-based biobank from Estonia. *Genet Med.* 2018.
34. Sturm AC, Knowles JW, Gidding SS, et al. Clinical Genetic Testing for Familial Hypercholesterolemia: JACC Scientific Expert Panel. *J Am Coll Cardiol.* 2018;72(6):662-680.
35. Kalia SS, Adelman K, Bale SJ, et al. Recommendations for reporting of secondary findings in clinical exome and genome sequencing, 2016 update (ACMG SF v2.0): a policy statement of the American College of Medical Genetics and Genomics. *Genet Med.* 2017;19(2):249-255.
36. Richards S, Aziz N, Bale S, et al. Standards and guidelines for the interpretation of sequence variants: a joint consensus recommendation of the American College of Medical Genetics and Genomics and the Association for Molecular Pathology. *Genet Med.* 2015.
37. Green RC, Berg JS, Grody WW, et al. ACMG recommendations for reporting of incidental findings in clinical exome and genome sequencing. *Genet Med.* 2013;15(7):565-574.
38. Landrum MJ, Lee JM, Benson M, et al. ClinVar: public archive of interpretations of clinically relevant variants. *Nucleic Acids Res.* 2016;44(D1):D862-868.
39. Amendola LM, Jarvik GP, Leo MC, et al. Performance of ACMG-AMP Variant-Interpretation Guidelines among Nine Laboratories in the Clinical Sequencing Exploratory Research Consortium. *Am J Hum Genet.* 2016;98(6):1067-1076.
40. Rivera-Munoz EA, Milko LV, Harrison SM, et al. ClinGen Variant Curation Expert Panel experiences and standardized processes for disease and gene-level specification of the ACMG/AMP guidelines for sequence variant interpretation. *Hum Mutat.* 2018;39(11):1614-1622.
41. Clinical Domain Working Group: Cardiovascular Familial Hypercholesterolemia Variant Curation Expert Panel. https://www.clinicalgenome.org/working-groups/clinical-domain/cardiovascular-clinical-domain-working-group/familial-hypercholesterolemia-variant-curation-expert-panel/. Accessed December 2, 2018.
42. Iacocca MA, Chora JR, Carrie A, et al. ClinVar database of global familial hypercholesterolemia-associated DNA variants. *Hum Mutat.* 2018;39(11):1631-1640.
43. Virani SS, Woodard LD, Wang D, et al. Correlates of repeat lipid testing in patients with coronary heart disease. *JAMA Intern Med.* 2013;173(15):1439-1444.
44. Horne R, Weinman J, Hankins M. The Beliefs about Medicines Questionnaire: the development and evaluation of a new method for assessing the cognitive representation of medication. *Psychol Health.* 1999;14(1):1-24.
45. Fowles JB, Terry P, Xi M, Hibbard J, Bloom CT, Harvey L. Measuring self-management of patients' and employees' health: Further validation of the Patient Activation Measure (PAM) based on its relation to employee characteristics. *Patient Educ Couns.* 2009;77(1):116-122.
46. Selim AJ, Rogers W, Fleishman JA, et al. Updated U.S. population standard for the Veterans RAND 12-item Health Survey (VR-12). *Qual Life Res.* 2009;18(1):43-52.
47. Behavioral Risk Factor Surveillance System. Centers for Disease Control and Prevention. http://www.cdc.gov/brfss/annual_data/pdf-ques/2009brfss.pdf. 2009.

VA Central IRB
Effective Date: April 26, 2022

48. Riordan F, McGann R, Kingston C, et al. A systematic review of methods to assess intake of saturated fat (SF) among healthy European adults and children: a DEDIPAC (Determinants of Diet and Physical Activity) study. *BMC Nutrition.* 2018;4(1):21.
49. Topolski TD, LoGerfo J, Patrick DL, Williams B, Walwick J, Patrick MB. The Rapid Assessment of Physical Activity (RAPA) among older adults. *Prev Chronic Dis.* 2006;3(4):A118.
50. Vassy JL, Christensen KD, Schonman EF, et al. The impact of whole-genome sequencing on the primary care and outcomes of healthy adult patients: A pilot randomized trial. *Ann Intern Med.* 2017;167(3):159-169.
51. Sanders GD, Neumann PJ, Basu A, et al. Recommendations for Conduct, Methodological Practices, and Reporting of Cost-effectiveness Analyses: Second Panel on Cost-Effectiveness in Health and Medicine. *JAMA.* 2016;316(10):1093-1103.
52. Li M, Bennette CS, Amendola LM, et al. The Feelings About genomiC Testing Results (FACToR) Questionnaire: Development and Preliminary Validation. *Journal of genetic counseling.* 2019;28(2):477-490.
53. Lewis SJ, Olufade T, Anzalone DA, Malangone-Monaco E, Evans KA, Johnston S. LDL cholesterol levels after switch from atorvastatin to rosuvastatin. *Curr Med Res Opin.* 2018;34(10):1717-1723.
54. Ramsey SD, Willke RJ, Glick H, et al. Cost-effectiveness analysis alongside clinical trials II-An ISPOR Good Research Practices Task Force report. *Value Health.* 2015;18(2):161-172.
55. Barnett PG, Hong JS, Carey E, Grunwald GK, Joynt Maddox K, Maddox TM. Comparison of Accessibility, Cost, and Quality of Elective Coronary Revascularization Between Veterans Affairs and Community Care Hospitals. *JAMA cardiology.* 2018;3(2):133-141.

VA Central IRB
Effective Date: April 26, 2022

Million Veteran Program Return of Actionable Results – Familial Hypercholesterolemia (MVP-ROAR-FH) Study

Statistical Analysis Plan

Funding Agency: Department of Veterans Affairs Office of Research and Development, Million Veteran Program

Principal Investigator: Jason L. Vassy, MD, MPH, MS
Biostatistician: Charles Brunette, PhD
Version Number 1.0

September 19, 2023

Role	Name	Signature	Date
Principal Investigator	Jason Vassy, MD	JASON VASSY 619380 (affiliate) Digitally signed by JASON VASSY 619380 (affiliate) Date: 2023.09.21 07:57:49 -04'00'	
Biostatistician	Charles Brunette, PhD	CHARLES BRUNETTE Digitally signed by CHARLES BRUNETTE Date: 2023.09.20 19:54:55 -05'00'	

List of Abbreviations

ADI	Area deprivation index
AE	Adverse event
apoB	Apolipoprotein (B)
ASCVD	Atherosclerotic cardiovascular disease
BMQ	Beliefs about Medicines Questionnaire
CDW	Corporate data warehouse
CIRB	(VA) Central Institutional Review Board
CMS	Centers for Medicare and Medicaid Services
DNA	Deoxyribonucleic acid
EHR	Electronic health record
FACToR	Feelings About Genomic Testing Results Questionnaire
FH	Familial hypercholesterolemia
IQR	Interquartile range
ITT	Intention-to-treat
LDL-C	Low-density lipoprotein cholesterol
LDLR	Low-density lipoprotein receptor gene
Lp(a)	Lipoprotein (a)
MCS	Mental component score (MCS)
MPR	Medication possession ratio
MVP	Million Veteran Program
MVP-ROAR-FH	Million Veteran Program Return of Actionable Results – Familial Hypercholesterolemia
N	Number of observations / participants
PAM-13	Patient Activation Measure - 13
PCS	Physical component score (VR-12)
PDC	Proportion of days covered
PI	Principal investigator
PCP	Primary care provider
RCT	Randomized controlled trial
SAE	Serious adverse event
SD	Standard deviation
VA	Veterans Affairs
VR-12	Veterans RAND 12-Item Health Survey

TABLE OF CONTENTS

1.0 ADMINISTRATIVE INFORMATION 4

2.0 INTRODUCTION 4

3.0 TRIAL METHODS 5

4.0 TRIAL POPULATION 8

5.0 STATISTICAL CONSIDERATIONS 11

6.0 OUTCOME DEFINITIONS AND TIMING 12

7.0 ANALYSIS METHODS 15

8.0 REFERENCES 18

1.0 Administrative Information

1.1 Trial title and registration

Million Veteran Program Return of Actionable Results - Familial Hypercholesterolemia (MVP-ROAR-FH) Study. ClinicalTrials.gov Identifier: NCT04178122

1.2 Revision history

SAP version	Section changed	Description	Date amended
1.0		Date created	09/19/2023

1.3 Key personnel

1.3.1 Principal investigator

The principal investigator (PI) supervises all aspects of the study. The PI takes responsibility for the scientific development and conduct of the study, including meeting study goals and timelines, monitoring participant safety, and oversight of the dissemination of research findings.

1.3.2 Biostatistician

The biostatistician advises the study team on the appropriate study design and statistical analysis of study outcomes. The biostatistician conducts and/or reviews sample size and power calculations and provides supervision to the data analyst in performing data collection, data cleaning, and statistical analysis of study data.

1.3.3 Data manager/analyst

The data manager creates and maintains the database housing study data. The data analyst ensures the capture of study data and performs requisite merging and cleaning of study data. The data manager prepares summary data tables for study planning, reporting, monitoring, and dissemination of results. The data manager prepares and maintains participant randomization tables and mechanisms for treatment allocation. The data manager does not engage in the enrollment or allocation of participants to study treatments. Under the direction of the biostatistician, the data analyst may perform statistical analysis of study outcomes.

1.3.4 Research genetic counselor / project manager

The research genetic counselor / project manager is responsible for the day-to-day operations of the MVP-ROAR-FH Study. They also obtain participant consent for study participation and genetic confirmation testing and deliver the intervention to enrolled participants.

2.0 Introduction

This document details the proposed data analysis, presentation, and reporting of outcomes associated with the MVP-ROAR-FH Study. The results reported in the primary study

manuscript(s) will adhere to the strategy outlined here. All amendments to this plan will be documented and reviewed by the relevant key personnel listed within this document. Any deviations to this plan will be justified and detailed in the final manuscript(s). Further analysis, including subset and exploratory analyses not included here, may occur as needed and will be justified and described if reported. This document follows the published guidelines for the content of statistical analysis plans in clinical trials.[1]

2.1 Background

Familial hypercholesterolemia (FH) is a genetic disorder characterized by elevated low-density lipoprotein cholesterol (LDL-C) levels and increased risk for cardiovascular disease. The MVP-ROAR-FH study aims to return potentially actionable genetic results associated with FH to Million Veteran Program (MVP) participants and their primary care providers. Informing participants of this information might lead to earlier interventions and improved health outcomes.

2.2 Study objectives

The primary objective of MVP-ROAR-FH is to evaluate the difference in change in LDL-C (end-of-study LDL-C minus baseline LDL-C) after 6 months between the Immediate Results arm and the Delayed Results arm. Secondary and exploratory objectives include an assessment of the difference in the proportions of participants reaching LDL-C targets between arms, examination of the difference in the intensification of lipid-lowering pharmacotherapy between arms, and evaluation of medication adherence, cascade testing, lifestyle behaviors, healthcare costs, and quality-of-life after a 6-month observation period.

2.3 Primary outcome and research hypothesis

Primary outcome: Change in LDL-C 6 months after randomization

Null hypothesis: 6-month change in LDL-C is not significantly different between groups receiving their genetic results at baseline compared to those receiving their results after 6 months.

Alternative hypothesis: 6-month change in LDL-C is significantly different between groups receiving their genetic results at baseline compared to those receiving their results after 6 months.

3.0 Trial Methods

3.1 Trial design

The MVP-ROAR-FH Study is a randomized controlled trial (RCT). MVP participants suspected of carrying a genetic variant associated with familial hypercholesterolemia are recontacted, enrolled, and, upon receipt of a DNA sample, randomly assigned either to an Immediate Results arm, where the return of genetic results intervention occurs at baseline, or a Delayed Results arm, where the intervention is delivered at the end of the study, after 6 months. The intervention consists of variant confirmation testing and reporting, standard genetic counseling, provision of informational resources to the participant and their primary care providers (PCPs), and documentation of the intervention in the medical record.

3.2 Sample size

The MVP-ROAR-FH Study aims to enroll ten participants into a pilot trial and 244 participants into the RCT.

Sample size is based on the primary outcome of change in LDL-C in each arm after 6 months. Assuming a mean LDL-C reduction of 20% in the Immediate Results arm, a mean LDL-C reduction of 0% in the Delayed Results arm, and a common standard deviation of 30%,[2] 72 total participants (36 per arm) are needed to have 80% power to detect a significant between-group difference at alpha=0.05. Enrollment of twice this number (144 total) will account for an absence of therapy escalation in up to 50% of participants in the Immediate Results arm. Enrollment of 180 total participants will account for up to 20% loss to follow-up.

**Change in LDL-C at 6 months**			**Total sample size required**
**Immediate Results**	**Delayed Results**	**Common SD**	
-20%	0%	30%	72
-20%	0%	40%	126
-20%	-5%	30%	126
-20%	-5%	40%	224

An important secondary outcome is the proportion of participants in each arm meeting accepted LDL-C targets at 6 months (<100 mg/dL for primary prevention and <70 mg/dL for secondary prevention). In preparatory-to-research analyses, only 175/322 (46%) MVP participants with a potentially pathogenic LDLR variant had a most recent LDL-C <100mg/dL. To have 80% power to detect a between-arm difference of 20% of participants meeting this LDL-C target at alpha=0.05, up to 194 total participants are needed (97 per arm). To account for up to 20% loss to follow-up, a total of 244 participants (122 per arm) are needed.

**Proportion of participants with LDL-C < 100mg/dL at 6 months**		**Total sample size required**
**Immediate Results**	**Delayed Results**	
10%	30%	124
20%	40%	162
30%	50%	186
40%	60%	194

3.3 Randomization

Study staff use pre-generated randomization tables for 1:1 allocation of participants to each study arm using a permuted block design with a block size of four. Pre-generated randomization tables are created using standard statistical software (*e.g.* computerized random block and sequence generation) by the MVP-ROAR-FH Study data manager, under the direction of the

biostatistician, and stored in a secure file share accessible to select study staff. Randomization occurs upon the completion of baseline procedures and confirmed receipt of each participant's DNA specimen and is mechanized through a computerized randomization tool. Study staff enrolling and allocating participants to study treatments are blinded to the pre-generated randomization tables.

3.4 Data sources, collection, and storage

Study outcomes data will be collected from the VA Corporate Data Warehouse (CDW), a repository of administrative and clinical data from the VA's nationally deployed electronic health record (EHR) system;[3] clinical chart review of the EHR, participant baseline and follow-up surveys, clinical confirmation genetic testing, and trial operations data recorded by the study team. All study data will be stored, cleaned, and analyzed within a secure VA computing environment and will be accessible to authorized study staff only.

3.5 Stopping guidance

This study has no stopping rules. Enrolled participants may withdraw from the study at any time.

3.6 Protocol deviations

Protocol deviations are characterized as circumstances that depart from planned study procedures and anticipated events (*e.g.*, participant withdrawal, loss to follow-up). Protocol deviations may include, but are not limited to, the following:

1. Deviation from inclusion or exclusion criteria (*e.g.*, ineligible patient enrolled and/or randomized)
2. Patient receipt of treatment other than treatment as randomized

The number of ineligible patients randomized, patients receiving a treatment other than as randomized, or other yet to be determined protocol deviations, if any, will be characterized and reported in the final manuscript(s). For the purposes of primary and secondary outcomes, data from patients who experience a protocol deviation will be included in the final data sets. Their outcomes data will be analyzed as part of the treatment group to which they were randomly allocated. The inclusion or exclusion of these patients' data in subsequent secondary or subgroup analyses will be detailed in the final manuscript(s) as needed.

3.7 Adverse events

Adverse events (AEs) related to MVP-ROAR-FH procedures do not include anticipated events related to blood draws (e.g., pain, minor bleeding, bruising, fainting, or lightheadedness) and minor feelings of discomfort while answering survey questions. Pre-existing conditions or illnesses which are expected to exacerbate or worsen are also not considered adverse events and will be accounted for in the subject's medical history. An AE may be considered any other unanticipated or unintended medical occurrence or worsening of a sign or symptom (including an abnormal laboratory finding other than the return of genetic information associated with FH) or disease in a study subject, which does not necessarily have a causal relationship with the study condition, procedure(s) or study agent(s), that occurs after participant informed consent is obtained. A serious adverse event (SAE) will be defined as an AE resulting in one of the following outcomes: death during the 6 months after study enrollment, life threatening event

(defined as an event that places a participant at immediate risk of death), inpatient hospitalization, and any other condition which, in the judgment of the PI, represents a significant hazard, such as an important medical event that does not result in one of the above outcomes. An event may be considered an SAE when it jeopardizes the participant or requires medical or surgical intervention to prevent one of the outcomes listed above. AEs may be observed by the study staff or volunteered by participants, their family members, their PCPs, or others. All AEs and SAEs will be assessed for relationship to the study research procedures by the study PI, to determine whether study participation was likely to have caused the AE/SAE.

4.0 Trial Population

The overall study population includes all participants of the Million Veteran Program (MVP) mega-biobank research study.

4.1 Study inclusion and exclusion criteria

Inclusion criteria: A participant is eligible for enrollment in this study if he/she meets the following criteria:

- Is a living enrollee in MVP.
- Is identified to have a pathogenic or likely pathogenic variant in an FH-associated gene in their MVP genotype data.
- Has not previously undergone genetic testing for familial hypercholesterolemia. Study staff first ascertain this by review of the medical record and then confirm during the informed consent call by asking the participants about any prior genetic testing he/she has undergone.
- Is not incarcerated.
- Is not pregnant.

4.2 Screening, recruitment, and withdraw

MVP Core study staff queries MVP databases for living participants with an eligible FH variant. The MVP Core study team mails eligible participants a letter introducing this new MVP-related study giving participants the opportunity to opt out of further contact by returning a prepaid opt out postcard or by calling the MVP Call Center. To any participant who does not opt out within 2 weeks of this initial mailing, the MVP-ROAR-FH study team mails a letter providing more detail about the study, including all necessary informed consent information. Two weeks after this mailing, the study genetic counselor calls the participant to review the informed consent information, answers any questions about the study, and documents verbal consent or decline. See MVP-ROAR-FH Study protocol VA CIRB 19-11 for additional detail regarding the study recruitment process.

Duration of the study recruitment period, the total number of patients screened, the number of screened patients not recruited and reason for non-recruitment, and other screening and recruitment metrics will be collected and reported for the overall study. In addition to protocol deviations and AEs or SAEs, if any, the number, and reasons (if known) for participant withdrawal and/or loss to follow-up prior to the conclusion of the study's period of enrollment will be reported in the final manuscript(s). Participant flow will be reported in the final manuscript

using CONSORT guidelines for the reporting of clinical trials.[4,5]

4.3 Reference start and end dates

Participant study enrollment occurs on the date of consent. Randomization occurs upon completion of baseline procedures (i.e. baseline survey) and confirmed receipt of DNA biospecimen (blood or saliva). Study participation concludes upon completion of the 6-month biospecimen collection and end-of-study survey, conducted approximately 6 months after the date of randomization and end-of-study survey.

4.3.1 Baseline assessment

For the purposes of outcomes assessment, baseline is defined as the most recent measurement of a study-related outcome (see Section 6.0) on or prior to a participant's date of randomization, unless otherwise specified. The total number of participants with baseline measurements obtained post-randomization and/or any statistical analysis including a baseline measurement obtained post-randomization will be reported.

4.3.2 End-of-study follow-up and period of observation

Enrolled participants will be observed for a total of 6 months. For the primary (change in LDL-C) and secondary (proportion meeting LDL-C targets) outcomes related to LDL-C, the end-of-study date will correspond to the date 6 months from the date of randomization. The most recent LDL-C value on or after this date, either associated with a completed study-related blood draw or LDL-C value extracted from CDW data (if unable to obtain an end-of-study specimen), will be used as the 6-month LDL-C value.

Similarly, for the secondary outcome associated with intensification of pharmacotherapy, the end-of-study date will correspond to the date 6 months from the date of randomization. All lipid-lowering related prescriptions, either derived from participant survey responses or extracted from CDW data, which are identified as active prior to or after the date of randomization and considered active either on or before the date 6 months from the date of randomization will be considered for analysis. Prescriptions are defined as active if they fall within a time window of one and a half times the total days supply (equivalent to a medication possession ratio of 0.67) from the prescription start date.[6–8]

4.4 Analysis populations

4.4.1 Intention-to-treat populations

Intention-to-treat (ITT) populations include all patients who undergo randomization and are characterized by the treatment they were randomized to receive (Immediate Results vs. Delayed Results).

4.4.2 Complete case populations

The complete case populations consist of patients who undergo randomization and complete all study assessments, including both the baseline and end-of-study patient surveys and end-of-study LDL-C measurement.

4.4.3 Subgroup populations

Additional subgroups of the study population may include the analyses of patients stratified by demographic (e.g. age), sex, or baseline LDL-C values. Each subgroup population will be described in detail as reported in the final study manuscript(s).

4.5 Baseline patient characteristics

Baseline characteristics will be summarized and presented for participants in the ITT populations. Standard statistical summaries, depending on data type and distribution, will be presented as 1) total numbers of participants with each characteristic (n) and as a proportion (%) of each group stratified by randomization arm or 2) as means and standard deviations (if normally distributed) or medians and interquartile ranges (IQR) (if non normally distributed) stratified by randomization arm. No statistical testing will be carried out for participant baseline characteristics or measures between treatment groups.

At minimum, the below participant baseline characteristics, including the pre-specified baseline measurements of the study outcomes described in Section 6.0, will be derived and reported:

**Baseline characteristic**	**How derived**	**Presentation**
Age in years	As calculated using EHR administrative data relative to date of consent and enrollment.	mean (SD) / median (IQR)
Gender / Sex	As determined by EHR administrative data.	n (%)
Race	As determined by EHR administrative data and/or data collected from baseline survey.	n (%)
Ethnicity	As determined by EHR administrative data and/or data collected from baseline survey.	n (%)
Socioeconomic Status / Area Deprivation Index	Calculated using income, geographic, and other EHR administrative data.(ADI)[9]	n (%)
Self-reported health status and quality of life	As determined by data collected from baseline survey. (VR-12)[10–12]	mean (SD) / median (IQR)
Self-reported patient activation	As determined by data collected from baseline survey. (PAM-13)[13]	mean (SD) / median (IQR) / n (%)
Beliefs about medications	As determined by data collected from baseline survey. (Beliefs About Medicines Questionnaire)[14]	mean (SD) / median (IQR)
Low-density lipoprotein cholesterol	As determined by EHR data.	mean (SD) / median (IQR)
Lipid-lowering pharmacotherapy	As determined by data collected from baseline survey and EHR data.	n (%)

5.0 Statistical Considerations

5.1 Statistical framework

The principal analysis uses an intention-to-treat (ITT)[15] approach to compare the Immediate Results and Delayed Results arms. Using an independent t-test, analysis of the primary outcome uses a t-statistic, and two-sided type I error rate of 0.05 to test the null hypothesis of no difference in LDL-C change between arms. To quantify the treatment arm difference for the primary endpoint, a mean difference will be presented with a corresponding confidence interval estimate. Mean change in LDL-C is also reported separately for each arm. Similarly, for secondary and other pre-specified outcomes an ITT approach is used to make outcomes comparisons across treatment groups. Subset analyses, including sensitivity and group analyses by demographic, variant confirmation status, or other study or patient characteristics, are considered exploratory and will be described in detail if reported.

5.2 Interim analyses

No formal interim hypothesis testing is planned.

5.3 Timing of final analyses

Final analyses of the MVP-ROAR-FH Study data are conducted upon the conclusion of the final participant's data collection procedures (*i.e.*, end-of-study survey and biospecimen collection).

5.4 Confidence intervals, *P* values, and multiple testing

All statistical testing is reported with an effect, a two-sided 95% confidence interval, and *P* value, unless otherwise specified. *P* values less than 0.05 will be reported as significant for the primary outcome. *P* values reported as significant for secondary outcomes will undergo Bonferroni correction for multiple hypothesis-testing. Other pre-specified and post-hoc analyses are considered exploratory.

5.5 Missing data

Prior to statistical analysis, outcomes data are reviewed for the amount and pattern of data missingness (*e.g.*, missing at random) using standard statistical software and methods. For outcomes analysis, partially observed outcomes may be imputed using mean or median imputation, multiple imputation, or comparable methods, as appropriate.[16–18] Any necessary imputation will be conducted separately within each treatment arm. Proportions of data missingness, reasons for missingness (if known), and methods used for data imputation if required, including number of imputations and sensitivity analyses performed, will be reported in the final manuscript(s).

5.6 Statistical assumptions and issues

Prior to analysis, statistical assumptions are evaluated for each proposed outcome assessment. The presence of distributional assumptions, influential outliers, and homogeneity of variance, among other common assumptions related to the analyses described here, are assessed. Methods used and results of the assessment of statistical assumptions will be

acknowledged in the final study manuscript(s). In addition, issues related to significant differences between withdrawn, lost to follow-up, and remaining cases as well as changes in study methods over time (e.g. change in study procedures that result in materially different patient outcomes) will be considered. A description of unusual outliers, violated assumptions, or other issues that may impact the integrity of the analyses, and any corrective action (e.g. assumptions evaluated, review of outliers/sensitivity analyses, variable transformations, etc.) will be described in the final study manuscript(s) as applicable.

5.7 Clustered data

Given the small number of participants relative to the number of VA PCPs and facilities nationally, there is little potential for clustering effect among patients receiving care from the same PCPs or at the same VA facility. As a result, provider or facility clustering will not be considered in the final analyses.

5.8 Statistical software

Statistical analysis will be conducted using appropriate and validated software, including SAS, STATA, R, or other comparable statistical programs. The applicable software(s), package(s), and version(s) used for the analyses of study data will be reported in the final manuscript(s).

6.0 Outcome definitions and timing

6.1 Primary outcome (6-month difference in LDL-C)

Baseline (at enrollment) and end-of-study (at 6 months) LDL-C values are defined as the LDL-C measurements obtained from study-related blood draws as processed by the study's centralized laboratory. For participants for whom it is not feasible to visit their local VA facility for a blood draw at baseline, a documented LDL-C value in their medical record may be used for a study measurement, provided it is not older than 6 months from their date of enrollment and a change in lipid-lowering therapy has not occurred between the clinical LDL-C value and study enrollment. For participants for whom it is not feasible to visit their local VA facility for an end-of-study blood draw, a documented LDL-C value in their medical record may be used for a study measurement. The documented LDL-C value closest to the date 6 months after the date of randomization will be used for the end-of-study value. If no 6-month value is available, the study may use an LDL-C value obtained from the medical record during the 6-month observation period, including carry forward of the baseline value.

6.2 Secondary outcomes

6.2.1. Proportion meeting LDL-C targets

The proportion of participants meeting individualized clinically significant LDL-C targets (<100mg/dL for primary prevention and <70 mg/dL for secondary prevention) will be determined at 6 months, using the LDL-C values as described for the primary outcome in Section 6.1.

Secondary prevention will be defined as any patient with any of the following:

Pre-existing atherosclerotic disease (ASCVD): acute coronary syndrome in the prior 12 months, history of myocardial infarction, history of ischemic stroke, symptomatic peripheral artery disease including aneurysm, all of atherosclerotic origin.

Presence of any ASCVD risk factors: age ≥65 years; prior percutaneous coronary intervention; prior coronary artery bypass graft; other evidence of coronary artery disease; diabetes mellitus; hypertension; chronic kidney disease; current smoking; congestive heart failure; family history of premature ASCVD; elevated coronary artery calcium score, Lp(a), or apoB; or ankle-brachial index <0.9.

All other participants will be considered eligible for primary prevention.

The primary or secondary prevention status of each participant will be determined by clinician review of participant medical records through the end-of-study date. The clinician reviewer(s) will be blinded to randomization status. For each participant deemed eligible for secondary prevention, the clinician will record the reason(s) for eligibility in a study database.

6.2.2. Intensification of pharmacotherapy

The proportion of participants with an intensification of lipid-lowering pharmacotherapy will be determined from baseline and end-of-study prescription data, obtained from the CDW, medical record review, and patient surveys. Intensification of pharmacotherapy at 6-months will be a composite outcome including prescription of new monotherapy, dose escalation of existing pharmacotherapy, or addition of one or more medications to existing pharmacotherapy compared to baseline pharmacotherapy status.

6.3 Other prespecified outcomes

6.3.1. Medication adherence

Continuous medication adherence is assessed using medication possession ratios (MPRs) or proportion of days covered (PDC) as derived from CDW pharmacy data.[6–8] All lipid-lowering related prescriptions, which are identified as active after the date of randomization and considered active either on or before the date 6 months from the date of randomization will be considered for analysis. PDC is calculated as the percentage of days in which a participant has access to a prescribed medication over the period of observation (assessed as sum of total days supply / total days in observation period). A measure of medication adherence is derived for each participant with a lipid-lowering medication prescription during the study observation period between the date of randomization and the date 6 months after randomization. Participants with a PDC of 80% or greater associated with lipid-lowering medications will be considered adherent. Total numbers of participants with lipid-lowering prescriptions, as well as subsets of participants with PDC measures will be reported in the final manuscript(s).

6.3.2. Cascade testing

The number of first-degree relatives undergoing genetic testing within 6 months will be recorded for each participant, as measured on the end-of-study survey.

6.3.3. Self-reported quality of life

Self-reported quality of life is assessed via the baseline and end-of-study surveys, using the Veterans RAND 12-Item Health Survey (VR-12).[10–12] The VR-12 computes two continuous composite scores, a physical component summary (PCS) and a mental component summary (MCS).

6.3.4. Lifestyle behaviors

Lifestyle behaviors are assessed using items structured on the transtheoretical model of behavior change.[19] Response options seek to assess at what point participants are in the behavior change process at 6 months. Response options assess readiness for change across myriad lifestyle behaviors using response options ranging from engaging in a specific behavior for more than 6 months (maintenance stage) to not at all (precontemplation stage).

a) Self-reported physical activity[20]

Self-reported physical activity is assessed with the baseline and end-of-study surveys, using the single-item question “Do you exercise 3 times a week for at least 20 minutes each time?”

b) Self-reported smoking status[21,22]

Self-reported smoking status is assessed with the baseline and end-of-study surveys, using two items: “Are you currently a smoker?” and, if yes, “Are you seriously thinking of quitting smoking?”

c) Self-reported saturated fat consumption[19]

Self-reported saturated fat consumption is assessed with the baseline and end-of-study surveys, using the single-item question “Do you consistently avoid eating high fat foods?”

6.3.5. Healthcare costs and utilization

A combination of administrative data, survey data, and microcosting approaches are used to estimate costs over the 6 months after randomization. Utilization of healthcare services, including laboratory tests, office visits, time demands, transportation cost, and hospitalization information will be derived from both end-of-study participant surveys and CDW administrative data.[23,24] Estimates of the infrastructure and personnel needed to deliver the intervention are derived empirically from the study. Healthcare costs are abstracted from billing and administrative data from the CDW and CMS data.

6.4 <u>Other exploratory outcomes</u>

6.4.1. Self-reported patient activation

Self-reported understanding, competence, and willingness to participate in health care decisions and processes are assessed via the baseline and end-of-study surveys, using the 13-item short form of the Patient Activation Measure (PAM-13).[13] Each PAM-13 item has four possible response options: “*Strongly disagree*” (1), “*Disagree*” (2), “*Agree*” (3), “*Strongly agree*” (4), as well as “*Does not apply*” (0). Response values are summed, divided by the total number of items responded to (excluding selections of non-applicable items), and multiplied by 13. The raw score is converted using a scoring table to derive both a linear score from 0 (no activation) to 100 (fully activated) and interval patient activation scores (1: activation not important, passive recipient of care; 2: lack of knowledge or confidence to take action; 3: beginning to take action; 4 taking action).

6.4.2. Beliefs about medications

Beliefs about medications is assessed on the baseline and end-of-study surveys, using the 8-item Beliefs About Medicines Questionnaire (BMQ) - General Scale.[14] Each item has five possible response options: (1) *Strongly disagree*, (2) *Disagree*, (3) *Neither agree nor disagree*, (4) *Agree*, (5) *Strongly agree*. A total score reflecting overall beliefs is calculated as a numerical sum across participant responses ranging from 8 to 40. Higher scores represent stronger beliefs about medication overuse and harm. The general use subscale is calculated as a numerical sum using items 1, 3, 4, and 8 ranging from 4 to 20 and represents beliefs about medication overuse. The general harm subscale is calculated as a numerical sum using items 2, 5, 6, and 7 ranging from 4 to 20 and represents beliefs about medication harm. A measure of medication beliefs is derived for each participant at baseline and 6 months after randomization.

6.4.3. Self-reported feelings about genomic testing results

Self-reported feelings about the psychosocial impact of receiving genomic test results will be assessed using the Feelings About genomic Testing Results (FACToR) Questionnaire.[25] The FACToR is only administered to the Immediate Results arm.

6.4.4. Preferences for receiving genetic test results

Preferences for receiving genetic test results include two items, developed specifically for this study, to assess 1) preferred first contact for genetic test results (e.g., participant, participant's primary care provider) and 2) preferred provider to deliver results (e.g., research genetic counselor, participant's primary care provider). These items are only administered to the Immediate Results arm.

7.0 Analysis methods

7.1 Covariate adjustment

No prespecified covariate adjustment is planned. If any covariate adjustment is deemed necessary during analysis or used to improve the precision of estimates, rationale and methods will be fully described in the final manuscript(s).

7.2 Primary outcome

The difference in LDL-C reduction after 6 months between the Immediate Results arm and the Delayed Results arm will be assessed. The LDL-C values at baseline and 6 months will first be summarized descriptively for each arm. The change in LDL-C from baseline to 6 months will be calculated for each participant. An independent *t*-test will be employed to compare the mean LDL-C change between the two arms, assuming normally distributed data. If the change scores do not follow a normal distribution, a non-parametric alternative, the Wilcoxon rank-sum test, may be used. The effect size (Cohen's d), confidence intervals, and *P* value will be reported to determine the statistical significance of the observed difference. Additionally, potential confounders may be included in regression modeling to ensure the robustness of findings.

7.3 Secondary outcomes

7.3.1 Proportion meeting LDL-C targets

Proportions of patients meeting individualized cholesterol targets at end of study (yes or no for <100mg/dL for primary prevention and <70 mg/dL for secondary prevention) will be compared between the Immediate Results and Delayed Results arm using either a Z-test of proportions or

chi-squared test. In the event of small cell counts, we will employ Fisher's exact test to ensure the validity of the results. We will compute the relative risk and 95% confidence intervals to quantify the strength and direction of the association. Potential confounders may be adjusted for by using logistic regression. Frequencies and proportions of participants meeting LDL-C targets will be reported by randomization arm.

7.3.2 Intensification of pharmacotherapy

Proportion of patients with intensification of pharmacotherapy from baseline to end of study will be compared between the Immediate Results and Delayed Results arm using either a Z-test of proportions or chi-squared test. In the event of small cell counts, we will employ Fisher's exact test to ensure the validity of the results. We will compute the relative risk and 95% confidence intervals to quantify the strength and direction of the association. Potential confounders may be adjusted for by using logistic regression. Frequencies and proportions of the intensification of medications among participants will be reported by randomization arm. Exploratory analyses may compare participant medication trajectories between groups, including intensification, unchanged prescription status, or deintensification status at baseline, 3 months, and 6 months using a generalized linear model fit with generalized estimating equations.[26]

7.4 Other prespecified and exploratory outcomes

7.4.1 Medication adherence

Proportions of patients considered adherent to lipid-lowering medication prescriptions (PDC ≥ 80%) are compared between the Immediate Results and Delayed Results arm using either a Z-test of proportions or chi-squared test. In the event of small cell counts, we will employ Fisher's exact test to ensure the validity of the results. We will compute the relative risk and 95% confidence intervals to quantify the strength and direction of the association.

7.4.2 Cascade testing

The number of first-degree relatives undergoing genetic testing within 6 months will be recorded. Mean numbers and standard deviations of first-degree relatives tested will be calculated for each arm. A Poisson regression or negative binomial regression (in case of overdispersion) will be used to compare the rates of cascade testing between the two study arms. Potential confounders may be included in regression modeling to ensure the robustness of findings.

7.4.3 Self-reported quality of life

Mean change scores (6-month scores minus baseline scores) and standard deviations for both PCS and MCS of the VR-12 will be calculated for each arm. Independent samples t-tests will compare differences in mean change between the two arms for both PCS and MCS. Multivariable linear regression models (e.g. ANCOVA) may be fit to account for potential confounding variables.

7.4.4 Healthcare costs

Healthcare costs will be described using means and standard deviations for both arms. The differences in mean healthcare costs between the two groups will be evaluated using a generalized linear model with appropriate distributional assumptions (e.g., gamma for right skewed cost data). Additional covariates may be included to obtain more precise estimates or to adjust for potential confounding variables. Treatment effect is characterized by treatment versus control arm, presented as mean follow-up estimates with accompanying standard errors, 95% confidence intervals and *P*

values. In the presence of substantial missing data, mixed modeling or other repeated measures designs may be implemented.

7.4.5 Categorical and ordinal outcome measures

The following measures are compared between treatment groups among the ITT populations using standard methods for categorical data analysis:[27,28]

- Self-reported physical activity
- Self-reported smoking status
- Self-reported saturated fat consumption
- Self-reported patient activation (categorical score)
- Self-reported feelings about genomic testing results (Immediate Results arm only, items)
- Preferences for receiving genetic test results (Immediate Results arm only)

Frequency of end-of-study self-reported responses to study surveys, including categorical, ordinal, and Likert items, will be reported by treatment group (n, %). Binary logistic regression will be used to compare end-of-study dichotomous outcomes between treatment groups. To assess post- treatment ordered outcomes (e.g., lifestyle behaviors along the transtheoretical model continuum) between treatment arms, we will use ordinal logistic regression (e.g., cumulative logit model). Initial models will include treatment group assignment, and when available, baseline measures as covariates. Treatment effect is characterized by an odds ratio estimate presented with standard error, 95% confidence interval, and *P* value.

7.4.6 Continuous outcome measures

The following continuous measures are compared between treatment groups among the ITT populations using standard linear methods:[29,30]

- Self-reported patient activation (linear score)
- Self-reported beliefs about medications
- Self-reported feelings about genomic testing results (Immediate Results arm only, score)

Mean and standard deviation (or median and IQR, if applicable) of continuous scores associated with end-of-study self-reported responses to study surveys will be reported by treatment group. Analysis of covariance (ANCOVA) is used to compare continuous follow-up measures, including participant baseline measures, when available, and treatment group assignment. Treatment effect is characterized by treatment versus control arm, presented as mean follow-up estimates with accompanying standard errors, 95% confidence intervals and *P* values. In the presence of substantial missing data, mixed modeling or other repeated measures designs may be implemented.

7.5 Additional analyses

Further exploratory analyses using the methods described may be conducted for all study outcomes between treatment arms and across relevant subgroups. Inclusion of additional covariates in the models described or use of alternative statistical methods may be implemented to enhance model precision, to adjust for differences in baseline factors or multilevel characteristics, or to improve the integrity of the analyses (e.g., in the event of substantial missing data), among other reasons. To assess robustness of ITT analyses, analyses may be replicated within the relevant complete case populations. The addition of covariates or use of alternative methods to assess primary, secondary, and other outcomes may be considered and are supplemental to the prespecified analyses.

Additional exploratory analyses may be conducted to further examine study data or address research questions that arise during the conduct of the study. Any exploratory analyses or use of alternative methods will be justified and described in detail if reported.

8.0 References

1. Gamble C, Krishan A, Stocken D, et al. Guidelines for the Content of Statistical Analysis Plans in Clinical Trials. *JAMA*. 2017;318(23):2337-2343. doi:10.1001/jama.2017.18556
2. Lewis SJ, Olufade T, Anzalone DA, Malangone-Monaco E, Evans KA, Johnston S. LDL cholesterol levels after switch from atorvastatin to rosuvastatin. *Curr Med Res Opin*. 2018;34(10):1717-1723. doi:10.1080/03007995.2017.1421147
3. Price LE, Shea K, Gephart S. The Veterans Affairs's Corporate Data Warehouse: Uses and Implications for Nursing Research and Practice. *Nurs Adm Q*. 2015;39(4):311-318. doi:10.1097/NAQ.0000000000000118
4. Schulz KF, Altman DG, Moher D, the CONSORT Group. CONSORT 2010 Statement: updated guidelines for reporting parallel group randomised trials. *Trials*. 2010;11(1):32. doi:10.1186/1745-6215-11-32
5. Butcher NJ, Monsour A, Mew EJ, et al. Guidelines for Reporting Outcomes in Trial Reports: The CONSORT-Outcomes 2022 Extension. *JAMA*. 2022;328(22):2252-2264. doi:10.1001/jama.2022.21022
6. Steiner JF, Prochazka AV. The assessment of refill compliance using pharmacy records: Methods, validity, and applications. *J Clin Epidemiol*. 1997;50(1):105-116. doi:10.1016/S0895-4356(96)00268-5
7. Zimolzak AJ, Spettell CM, Fernandes J, et al. Early Detection of Poor Adherers to Statins: Applying Individualized Surveillance to Pay for Performance. *PLoS One*. 2013;8(11):e79611. doi:10.1371/journal.pone.0079611
8. Centers for Medicare and Medicaid Services. *Medicare 2023 Part C &D Star Ratings Technical Notes*.; 2023. Accessed September 1, 2023. https://www.cms.gov/files/document/2023-star-ratings-technical-notes.pdf
9. Kind AJH, Buckingham WR. Making Neighborhood-Disadvantage Metrics Accessible — The Neighborhood Atlas. *N Engl J Med*. 2018;378(26):2456-2458. doi:10.1056/NEJMp1802313
10. Jones D, Kazis L, Lee A, et al. Health Status Assessments Using the Veterans SF-12 and SF-36: Methods for Evaluating Outcomes in the Veterans Health Administration. *J Ambulatory Care Manage*. 2001;24(3):68-86. doi:10.1097/00004479-200107000-00011
11. Kazis LE, Selim A, Rogers W, Ren XS, Lee A, Miller DR. Dissemination of methods and results from the veterans health study: final comments and implications for future monitoring strategies within and outside the veterans healthcare system. *J Ambulatory Care Manage*. 2006;29(4):310-319. doi:10.1097/00004479-200610000-00007
12. Selim AJ, Rogers W, Fleishman JA, et al. Updated U.S. population standard for the Veterans RAND 12-item Health Survey (VR-12). *Qual Life Res*. 2009;18(1):43-52. doi:10.1007/s11136-008-9418-2
13. Hibbard JH, Mahoney ER, Stockard J, Tusler M. Development and Testing of a Short Form of the Patient Activation Measure. *Health Serv Res*. 2005;40(6p1):1918-1930. doi:10.1111/j.1475-6773.2005.00438.x
14. Horne R, Weinman J, Hankins M. The beliefs about medicines questionnaire: The development and evaluation of a new method for assessing the cognitive representation of medication. *Psychol Health*. 1999;14(1):1-24. doi:10.1080/08870449908407311
15. Lachin JM. Statistical Considerations in the Intent-to-Treat Principle. *Control Clin Trials*. 2000;21(3):167-189. doi:10.1016/S0197-2456(00)00046-5
16. Spiro A, Rogers W, Qian S, Kazis E. Imputing Physical and Mental Summary Scores (PCS and MCS) for the Veterans SF-12 Health Survey in the Context of Missing Data. In: *Technical*

Report, Scoring Algorithms and Users Guide, Submitted and Approved by the Center for Medicare and Medicaid Services (CMS) and National Committee for Quality Assurance (NCQA). ; 2004.
17. Lee KJ, Carlin JB. Multiple Imputation for Missing Data: Fully Conditional Specification Versus Multivariate Normal Imputation. *Am J Epidemiol*. 2010;171(5):624-632. doi:10.1093/aje/kwp425
18. Li P, Stuart EA, Allison DB. Multiple Imputation: A Flexible Tool for Handling Missing Data. *JAMA*. 2015;314(18):1966-1967. doi:10.1001/jama.2015.15281
19. Nigg CR, Burbank PM, Padula C, et al. Stages of change across ten health risk behaviors for older adults. *The Gerontologist*. 1999;39(4):473-482. doi:10.1093/geront/39.4.473
20. Marcus BH, Selby VC, Niaura RS, Rossi JS. Self-efficacy and the stages of exercise behavior change. *Res Q Exerc Sport*. 1992;63(1):60-66. doi:10.1080/02701367.1992.10607557
21. DiClemente CC, Prochaska JO, Fairhurst SK, Velicer WF, Velasquez MM, Rossi JS. The process of smoking cessation: an analysis of precontemplation, contemplation, and preparation stages of change. *J Consult Clin Psychol*. 1991;59(2):295-304. doi:10.1037//0022-006x.59.2.295
22. Velicer WF, Fava JL, Prochaska JO, Abrams DB, Emmons KM, Pierce JP. Distribution of smokers by stage in three representative samples. *Prev Med*. 1995;24(4):401-411. doi:10.1006/pmed.1995.1065
23. Sanders GD, Neumann PJ, Basu A, et al. Recommendations for Conduct, Methodological Practices, and Reporting of Cost-effectiveness Analyses: Second Panel on Cost-Effectiveness in Health and Medicine. *JAMA*. 2016;316(10):1093-1103. doi:10.1001/jama.2016.12195
24. Vassy JL, Christensen KD, Schonman EF, et al. The Impact of Whole-Genome Sequencing on the Primary Care and Outcomes of Healthy Adult Patients: A Pilot Randomized Trial. *Ann Intern Med*. 2017;167(3):159-169. doi:10.7326/M17-0188
25. Li M, Bennette CS, Amendola LM, et al. The Feelings About genomiC Testing Results (FACToR) Questionnaire: Development and Preliminary Validation. *J Genet Couns*. 2019;28(2):477-490. doi:10.1007/s10897-018-0286-9
26. Touloumis A. R Package multgee: A Generalized Estimating Equations Solver for Multinomial Responses. *J Stat Softw*. 2015;64:1-14. doi:10.18637/jss.v064.i08
27. Agresti A. *Categorical Data Analysis*. 3rd ed. Wiley-Interscience; 2013.
28. Harrell FE. *Regression Modeling Strategies With Applications to Linear Models, Logistic and Ordinal Regression, and Survival Analysis*. 2nd ed. Springer International Publishing; 2015.
29. Vickers AJ, Altman DG. Statistics notes: Analysing controlled trials with baseline and follow up measurements. *BMJ*. 2001;323(7321):1123-1124. doi:10.1136/bmj.323.7321.1123
30. O'Connell NS, Dai L, Jiang Y, et al. Methods for Analysis of Pre-Post Data in Clinical Research: A Comparison of Five Common Methods. *J Biom Biostat*. 2017;8(1):1-8. doi:10.4172/2155-6180.1000334
