# Supplementary material for: Opportunistic Genomic Screening for Familial Hypercholesterolemia to Improve Low-Density Lipoprotein Cholesterol: A Randomized Clinical Trial
Source: JAMA Netw Open. 2026 Jan 9;9(1):e2549664. doi: 10.1001/jamanetworkopen.2025.49664 (PMC12789956; doi:10.1001/jamanetworkopen.2025.49664)
Supplement: Supplement 2. — eTable 1. MVP-ROAR Study Enrollees Excluded From Per-Protocol Analyses or With Otherwise Atypical Results eTable 2. Per-Protocol Analyses of 6-Month LDL Cholesterol and Lipid-Lowering Therapy Outcomes Among MVP-ROAR Study Trial Participants eTable 3. Changes in Lipid-Lowering Therapy During 6-Month Observation Period Among MVP-ROAR Study Trial Participants eTable 4. Participant Responses to Receiving Genetic Results at 6 Months eTable 5. Baseline and 6-Month Patient-Reported Outcomes Among MVP-ROAR Study Participants eTable 6. Stages of Health Behavior Change Among MVP-ROAR Study Participants at 6 Months [file jamanetwopen-e2549664-s002.pdf]

# Supplementary Online Content

Vassy JL, Brunette CA, Yi T, et al; Veterans Affairs Million Veteran Program. Opportunistic genomic screening for familial hypercholesterolemia to improve low-density lipoprotein cholesterol: a randomized clinical trial. *JAMA Netw Open.* 2025;8(12):e2549664. doi:10.1001/jamanetworkopen.2025.49664

**eTable 1.** MVP-ROAR Study Enrollees Excluded From Per-Protocol Analyses or With Otherwise Atypical Results
**eTable 2.** Per-Protocol Analyses of 6-Month LDL Cholesterol and Lipid-Lowering Therapy Outcomes Among MVP-ROAR Study Trial Participants
**eTable 3.** Changes in Lipid-Lowering Therapy During 6-Month Observation Period Among MVP-ROAR Study Trial Participants
**eTable 4.** Participant Responses to Receiving Genetic Results at 6 Months
**eTable 5.** Baseline and 6-Month Patient-Reported Outcomes Among MVP-ROAR Study Participants
**eTable 6.** Stages of Health Behavior Change Among MVP-ROAR Study Participants at 6 Months

This supplementary material has been provided by the authors to give readers additional information about their work.

© 2025 Vassy JL et al. *JAMA Network Open*.

**eTable 1.** MVP-ROAR Study Enrollees Excluded From Per-Protocol Analyses or With Otherwise Atypical Results

| **Suspected variant from MVP array** | **Reason from per-protocol exclusion, if applicable** | **Gene panel results, if applicable** |
|---|---|---|
| NM_000527.4 *LDLR* 1055G>A | Testing not completed | - |
| NM_000527.4 *LDLR* 1055G>A | Testing not completed | - |
| NM_000527.4 *LDLR* c.1775G>A | Testing not completed | - |
| NM_000527.4 *LDLR* c.910G>A | Testing not completed | - |
| NM_000384.2 *APOB* c.10238del | FHBL | NM_000384.2 *APOB* c.10238del |
| NM_000384.2 *APOB* c.7537C>T | FHBL | NM_000384.2 *APOB* c.7537c>T |
| NM_000527.4 *LDLR* c.2000G>A | Research result not confirmed | Negative |
| NM_000527.4 *LDLR* c.2096C>T | Testing not completed | - |
| NM_000384.2 *APOB* c.10238del | FHBL | NM_000384.2 *APOB* c.10238del |
| NM_000527.4 *LDLR* c.2140+1G>A | Testing not completed | - |
| NM_000527.4 *LDLR* c.718G>A | Testing not completed | - |
| NM_000527.4 *LDLR* c.862G>A | Testing not completed | - |
| NM_000384.2 *APOB* c.10238del | FHBL | NM_000384.2 *APOB* c.10238del |
| NM_000527.4 *LDLR* c.296C>G | Research result not confirmed | Negative |
| NM_000527.4 *LDLR* c.1576C>T | Testing not completed | - |
| NM_000384.2 *APOB* c.10238del | FHBL | NM_000384.2 *APOB* c.10238del |
| NM_000384.2 *APOB* c.10238del | FHBL | NM_000384.2 *APOB* c.10238del |
| NM_000384.2 *APOB* c.10238del | FHBL | NM_000384.2 *APOB* c.10238del; NM_174936.3 *PCSK9* c.524+6G>A (VUS) |
| NM_000384.2 *APOB* c.10238del | FHBL | NM_000384.2 *APOB* c.10238del |
| NM_000527.4 *LDLR* c.621C>T | Research result not confirmed | Negative |
| NM_000527.4 *LDLR* c.1783C>T | Research result not confirmed | Negative |
| NM_000384.2 *APOB* c.10238del | FHBL | NM_000384.2 *APOB* c.10238del |
| NM_000527.4 *LDLR* c.862G>A | Testing not completed | - |
| NM_000527.4 *LDLR* c.296C>G | Research result not confirmed; found to have different *LDLR* variant and an *APOB* variant* | NM_000527.4 *LDLR* c.1586+1G>A; NM_000384.2 *APOB* c.11273T>C |
| NM_000527.4 *LDLR* c.296C>G | Research result not confirmed | Negative |
| NM_000384.2 *APOB* c.10238del | FHBL | NM_000384.2 *APOB* c.10238del |
| NM_000384.2 *APOB* c.10238del | FHBL | NM_000384.2 *APOB* c.10238del |
| NM_000384.2 *APOB* c.7537C>T | FHBL | NM_000384.2 *APOB* c.7537C>T |
| NM_000527.4 *LDLR* c.1055G>A | Research result not confirmed | Negative |
| NM_000384.2 *APOB* c.10238del | FHBL | NM_000384.2 *APOB* c.10238del |
| NM_000527.4 *LDLR* c.2000G>A | Research result not confirmed | Negative |
| NM_000384.2 *APOB* c.10238del | FHBL | NM_000384.2 *APOB* c.10238del |

*Included in per-protocol analysis. Reference is based on genome build GRCh37. Abbreviations: FHBL, Familial hypobetalipoproteinemia; LFU, lost to follow-up; VUS, variant of uncertain significance.

© 2025 Vassy JL et al. *JAMA Network Open*.

**eTable 2.** Per-Protocol Analyses of 6-Month LDL Cholesterol and Lipid-Lowering Therapy Outcomes Among MVP-ROAR Study Trial Participants

| LDL-C values, mean (SD), mg/dL | Overall (*n*=81) | Immediate Results arm (*n*=43) | Delayed Results arm (*n*=38) | Between-arm difference (95% CI) | Between-arm comparison |
|---|---|---|---|---|---|
| **Baseline LDL-C** | 120.1 (56.0) | 127.6 (64.4) | 111.6 (44.0) | 16.1 (-8.7, 40.8) | |
| **6-month LDL-C** | 117.3 (58.8) | 119.3 (70.7) | 115.0 (42.2) | 4.4 (-21.8, 30.5) | |
| **ΔLDL-C** | -2.8 (34.3) | -8.3 (35.9) | 3.4 (31.7) | -11.7 (-26.8, 3.4) | $p$=0.126[a]; d=0.34[b] |

Per-protocol analysis based on removal of non-confirmed and/or non-FH results (*n*=10), negative findings (*n*=7), and FHBL variants (*n*=14) and per-protocol analysis of one Delayed Results arm participant who received results immediately.
[a]Two-sample t-test (using absolute ΔLDL-C); similar results using ANCOVA (*p*=0.201) and Wilcoxon Rank Sum (*p*=0.115).
[b]Cohen's *d* effect size standards: small effect=0.2, medium effect=0.5, large effect=0.8.

© 2025 Vassy JL et al. *JAMA Network Open*.

**eTable 3.** Changes in Lipid-Lowering Therapy During 6-Month Observation Period Among MVP-ROAR Study Trial Participants

| | **Immediate arm (*n*=55[a])** | **Delayed arm (*n*=57)** | **Total (*n*=112)** |
|---|---|---|---|
| **Medication intensification, *n* (%)** | 11 (20%) | 5 (8.8%) | 16 (14.3%) |
| Initiation of treatment | 5 | 3 | 8 |
| Dose escalation | 2 | 1 | 3 |
| Additional medication | 5 | 1 | 6 |
| **No medication intensification, *n* (%)** | 44 (80%) | 52 (91.2%) | 96 (85.7%) |
| On treatment at baseline, no escalation | 26 | 37 | 63 |
| Not on treatment at baseline or follow-up | 13 | 11 | 24 |
| Treatment de-intensification, *n* (%) | 5 | 4 | 9 |

[a]One participant in the immediate arm had both a dose escalation and a medication addition (included in both counts)

© 2025 Vassy JL et al. *JAMA Network Open*.

**eTable 4.** Participant Responses to Receiving Genetic Results at 6 Months

| | **Immediate results (*n*=55)** |
|---|---|
| **Shared genetic result with at least one relative, *n* (%)**[a] | 30 (61%) |
| Total number of relatives with whom results were shared | 98 |
| **Reported that at least one relative underwent cascade genetic testing, *n* (%)**[a] | 3 (6.1%) |
| Total number of relatives with genetic testing | 4 |
| **Feelings about genomic test results (FACToR), mean (SD); 95% CI**[b] | |
| Negative emotions | 0.61 (1.53); 0.17, 1.05 |
| Positive feelings | 8.53 (3.53); 7.51, 9.54 |
| Uncertainty | 1.78 (2.08); 1.18, 2.37 |
| Privacy concerns | 0.22 (0.92); 0, 0.49 |
| **Preferences for receiving genetic test results: Who should the research team have contacted first about your genetic research results?, *n* (%)**[c] | |
| You | 35 (66%) |
| Your primary care provider | 9 (17%) |
| Unsure | 4 (8%) |
| Other | 4 (8%) |
| Does not apply | 1 (2%) |
| **After you got the clinical genetic testing to confirm the research result, who should have told you those results?, *n* (%)**[a] | |
| The genetic counselor on the research team | 25 (51%) |
| Your primary care provider | 12 (24%) |
| Unsure | 7 (14%) |
| Other | 5 (10%) |

[a]Results from 49 participants who completed these items. [b]FACToR scales range from 0 to maximum values in each category of feelings, where maximum value indicates greatest psychosocial impairment in that category. Maximum values for each category are: negative emotions, 12; positive feelings, 16; uncertainty, 12; privacy concerns, 8. Results shown are from 49 participants who completed any of this 12-item scale; additional 15 had missing responses from individual FACToR items, completed using median imputation. [c]Results from 53 participants who completed this item.

© 2025 Vassy JL et al. *JAMA Network Open*.

**eTable 5.** Baseline and 6-Month Patient-Reported Outcomes Among MVP-ROAR Study Participants

| | Overall (*n*=112) | Immediate (*n*=55) | Delayed (*n*=57) | Between-arm comparison[d] |
|---|---|---|---|---|
| **Health-related quality of life[a]** | | | | |
| Physical component | | | | |
| Baseline, mean (SD) | 38.70 (12.80) | 38.32 (11.05) | 39.08 (14.47) | |
| 6-month, mean (SD) | 39.83 (12.68) | 39.10 (11.14) | 40.58 (14.14) | |
| Change, mean (SD) | 1.14 (4.63) | 0.78 (4.45) | 1.50 (4.83) | *p*=0.38 |
| Mental component | | | | |
| Baseline, mean (SD) | 48.72 (13.46) | 47.78 (13.98) | 49.69 (12.98) | |
| 6-month, mean (SD) | 48.68 (13.71) | 47.38 (14.29) | 50.00 (13.09) | |
| Change, mean (SD) | -0.05 (5.53) | -0.40 (6.29) | 0.31 (4.66) | *p*=0.43 |
| **Patient activation[b]** | | | | |
| Baseline | | | | |
| Mean (SD) | 65.75 (13.35) | 65.53 (13.19) | 65.97 (13.63) | |
| Level, *n* (%) | | | | |
| 1 | 6 (5.5%) | 3 (5.5%) | 3 (5.5%) | |
| 2 | 19 (17.3%) | 9 (16.4%) | 10 (18.2%) | |
| 3 | 44 (40.0%) | 22 (40.0%) | 22 (40.0%) | |
| 4 | 41 (37.3%) | 21 (38.2%) | 20 (36.4%) | |
| 6-month | | | | |
| Mean (SD) | 63.31 (13.42) | 62.28 (11.65) | 64.33 (15.02) | |
| Level, *n* (%) | | | | |
| 1 | 8 (7.3%) | 3 (5.5%) | 5 (9.1%) | |
| 2 | 27 (24.5%) | 14 (25.5%) | 13 (23.6%) | |
| 3 | 48 (43.6%) | 25 (45.5%) | 23 (41.8%) | |
| 4 | 27 (24.5%) | 13 (23.6%) | 14 (25.5%) | |
| Change | | | | |
| Mean change (SD) | -2.44 (12.44) | -3.24 (13.05) | -1.63 (11.87) | *p*=0.40 |
| Change in level, median (IQR) | 0 (1) | 0 (1) | 0 (1) | *p*=0.99 |
| **Beliefs about medications[c]** | | | | |
| Baseline | 21.40 (5.03) | 21.33 (5.59) | 21.48 (4.44) | |
| Harm subscale | 8.51 (2.61) | 8.55 (2.86) | 8.48 (2.36) | |
| Overuse subscale | 12.89 (3.18) | 12.78 (3.41) | 13.00 (2.96) | |
| 6-month | 20.85 (5.14) | 21.31 (5.71) | 20.39 (4.49) | |
| Harm subscale | 8.35 (2.61) | 8.42 (2.78) | 8.28 (2.45) | |
| Overuse subscale | 12.50 (3.20) | 12.89 (3.59) | 12.11 (2.72) | |
| Change | -0.55 (3.25) | -0.01 (3.47) | -1.09 (2.94) | *p*=0.08 |
| Harm subscale | -0.17 (1.76) | -0.13 (1.82) | -0.20 (1.72) | *p*=0.78 |
| Overuse subscale | -0.39 (2.39) | 0.11 (2.47) | -0.89 (2.25) | *p*=0.03 |

Health-related quality of life measured by the Veterans RAND 12 Item Health Survey (VR-12) physical and mental components with ranges from 0 to 100, with a higher score indicating better health. Patient activation measured by the Patient Activation Measure (PAM-13), a 13-item measure to assess an individual's ability to manage their own health on a scale of 0-100, with a higher score indicating increased patient agency and proactive health behaviors. PAM-13 can also be categorized by levels of activation: Level 1: Disengaged and potentially overwhelmed (0-47.0); Level 2: Becoming aware but still struggling (47.1-55.1); Level 3: Taking action and gaining control (55.2-72.4); Level 4: The most active patients (72.5-100). The Beliefs About Medicines Questionnaire (BMQ) has a composite score with maximum 40, with higher scores indicating medications are generally overused and harmful, in addition to individual harm and overuse subscales. [a]Data fully missing from 3 participants in Delayed Results arm; 6-month data missing from 6 and 10 additional participants in the Immediate and Delayed Results arm, respectively. [b]Data fully missing from 2 participants in Delayed Results arm; missing responses on certain items from 6 and 10 additional participants in the Immediate and Delayed Results arm, respectively. [c]Data fully missing from 3 participants in Delayed Results arm; missing responses on certain items from 1 and 15 additional participants in the Immediate and Delayed Results arm, respectively. Imputation was done by replicating the corresponding baseline score of each individual. [d]*p*-values derived using Mann-Whitney test for ordinal data and analysis of covariance (ANCOVA) for continuous data.

© 2025 Vassy JL et al. *JAMA Network Open*.

**eTable 6.** Stages of Health Behavior Change Among MVP-ROAR Study Participants at 6 Months

| | Overall (*n*=112) | Immediate (*n*=55) | Delayed (*n*=57) | Between-arm comparison[c] |
|---|---|---|---|---|
| **6-month physical activity, *n* (%)[a]** | | | | *p*=0.73 |
| YES, I have been for MORE than 6 months | 57 (60.6%) | 30 (60.0%) | 27 (61.4%) | |
| YES, I have been, but for less than 6 months | 7 (7.4%) | 3 (6.0%) | 4 (9.1%) | |
| NO, but I intend to in the next 30 days | 10 (10.6%) | 4 (8.0%) | 6 (13.6%) | |
| NO, but I intend to in the next 6 months | 7 (7.4%) | 6 (12%) | 1 (2.3%) | |
| NO, and I do NOT intend to in the next 6 months | 13 (13.8%) | 7 (14.0%) | 6 (13.6%) | |
| **6-month smoking status, *n* (%)[a]** | | | | *p*=0.08 |
| Never smoked | 38 (40.4%) | 15 (30.0%) | 23 (52.3%) | |
| Quit > 6 months | 46 (48.9%) | 30 (60.0%) | 16 (36.4%) | |
| Quit within 6 months | 3 (3.2%) | 1 (2.0%) | 2 (4.5%) | |
| Current smoker | 7 (7.4%) | 4 (8.0%) | 3 (6.8%) | |
| **6-month saturated fat consumption, *n* (%)[b]** | | | | *p*=0.17 |
| YES, I have been for MORE than 6 months | 51 (55.4%) | 23 (47.9%) | 28 (63.6%) | |
| YES, I have been, but for less than 6 months | 2 (2.2%) | 2 (4.2%) | 0 (0.0%) | |
| NO, but I intend to in the next 30 days | 4 (4.3%) | 1 (2.1%) | 3 (6.8%) | |
| NO, but I intend to in the next 6 months | 3 (3.3%) | 3 (6.3%) | 0 (0.0%) | |
| NO, and I do NOT intend to in the next 6 months | 32 (34.8%) | 19 (39.6%) | 13 (29.5%) | |

[a]Data missing from 5 and 13 participants in immediate and delayed results arm, respectively. [b]Data missing from 7 and 13 participants from the immediate and delayed results arms, respectively. [c]P-values derived using Mann-Whitney test for ordinal data.

© 2025 Vassy JL et al. *JAMA Network Open*.
