# Supplementary material for: Opportunistic Genomic Screening for Familial Hypercholesterolemia to Improve Low-Density Lipoprotein Cholesterol: A Randomized Clinical Trial
Source: JAMA Netw Open. 2026 Jan 9;9(1):e2549664. doi: 10.1001/jamanetworkopen.2025.49664 (PMC12789956; doi:10.1001/jamanetworkopen.2025.49664)
Supplement: Supplement 3. — Nonauthor Collaborators [file jamanetwopen-e2549664-s003.pdf]

Supplemental Online Content: Nonauthor Collaborators
*First name, last name, and suffix (if applicable) are required and will appear in PubMed.

***Group Name(s): The Veterans Affairs Million Veteran Program**

***First Name and Middle Initial(s)**	***Last Name**	***Suffix (eg, Jr, III)**	Academic Degrees	Institution	Location (city, state/province, country)	Role or Contribution, eg, chair, principal investigator	Group (if more than 1 Group listed in the byline) and/or Subgroup (eg, Steering Committee)
Sumitra	Muralidhar		Ph.D.	US Department of Veterans Affairs	Washington, DC, USA	MVP Program Office	Million Veteran Program
Jennifer	Moser		Ph.D.	US Department of Veterans Affairs	Washington, DC, USA	MVP Program Office	Million Veteran Program
Jennifer E.	Deen		B.S.	US Department of Veterans Affairs	Washington, DC, USA	MVP Program Office	Million Veteran Program
Philip S.	Tsao		Ph.D.	VA Palo Alto Health Care System	Palo Alto, CA, USA	Principal investigator	Million Veteran Program
J. Michael	Gaziano		M.D., M.P.I	VA Boston Healthcare System	Boston, MA, USA	Principal investigator	Million Veteran Program
Elizabeth	Hauser		Ph.D.	Durham VA Medical Center	Durham, NC, USA	MVP Executive Commit	Million Veteran Program
Amy	Kilbourne		Ph.D., M.P.	VA HSR&D	Ann Arbor, MI, USA	MVP Executive Commit	Million Veteran Program
Michael	Matheny		M.D., M.S.,	VA Tennessee Valley Healthcare Syst	Nashville, TN, USA	MVP Executive Commit	Million Veteran Program
Dave	Oslin		M.D.	Philadelphia VA Medical Center	Philadelphia, PA, USA	MVP Executive Commit	Million Veteran Program
Deepak	Voora		M.D.	Durham VA Medical Center	Durham, NC, USA	MVP Executive Commit	Million Veteran Program
Jessica V.	Brewer		M.P.H.	VA Boston Healthcare System	Boston, MA, USA	MVP Core Operations	Million Veteran Program
Mary T.	Brophy		M.D., M.P.I	VA Boston Healthcare System	Boston, MA, USA	MVP Core Operations	Million Veteran Program
Kelly	Cho		M.P.H., Ph.	VA Boston Healthcare System	Boston, MA, USA	MVP Core Operations	Million Veteran Program
Lori	Churby		B.S.	VA Palo Alto Health Care System	Palo Alto, CA, USA	MVP Core Operations	Million Veteran Program
Scott L.	DuVall		Ph.D	VA Salt Lake City Health Care System	Salt Lake City, UT, USA	MVP Core Operations	Million Veteran Program
Saiju	Pyarajan		Ph.D	VA Boston Healthcare System	Boston, MA, USA	MVP Core Operations	Million Veteran Program
Robert	Ringer		Pharm.D	New Mexico VA Health Care System	Albuquerque, NM, USA	MVP Core Operations	Million Veteran Program
Luis E.	Selva		Ph.D	VA Boston Healthcare System	Boston, MA, USA	MVP Core Operations	Million Veteran Program
Shahpoor (Alex)	Shayan		M.S.	VA Boston Healthcare System	Boston, MA, USA	MVP Core Operations	Million Veteran Program
Brady	Stephens		M.S.	Canandaigua VA Medical Center	Canandaigua, NY, USA	MVP Core Operations	Million Veteran Program
Stacey B.	Whitbourne		Ph.D	VA Boston Healthcare System	Boston, MA, USA	MVP Core Operations	Million Veteran Program
