# Supplementary material for: Opportunistic Genomic Screening for Familial Hypercholesterolemia to Improve Low-Density Lipoprotein Cholesterol: A Randomized Clinical Trial
Source: JAMA Netw Open. 2026 Jan 9;9(1):e2549664. doi: 10.1001/jamanetworkopen.2025.49664 (PMC12789956; doi:10.1001/jamanetworkopen.2025.49664)
Supplement: Supplement 4. — Data Sharing Statement [file jamanetwopen-e2549664-s004.pdf]

Data Sharing Statement

Vassy. Opportunistic Genomic Screening for Familial Hypercholesterolemia to Improve Low-Density Lipoprotein Cholesterol. *JAMA Netw Open*. Published January 06, 2026. doi:10.1001/jamanetworkopen.2025.49664

Data
Additional Information: ClinicalTrials.gov ID NCT04178122
Data available: No

Additional Information
Explanation for why data not available: Million Veteran Program (MVP) and MVP Return Of Actionable Results (MVP-ROAR) participants did not consent to the sharing of individual-level data.
